# Loss of tau expression attenuates neurodegeneration associated with α-synucleinopathy

**DOI:** 10.1186/s40035-022-00309-x

**Published:** 2022-07-01

**Authors:** Scott C. Vermilyea, Anne Christensen, Joyce Meints, Balvindar Singh, Héctor Martell-Martínez, Md. Razaul Karim, Michael K. Lee

**Affiliations:** 1 Department of Neuroscience, University of Minnesota – Twin Cities, 321 Church Street S.E., Minneapolis, MN USA 6-145 Jackson Hall,; 2 Institute for Translational Neuroscience, University of Minnesota – Twin Cities, Minneapolis, MN USA; 3 Aligning Science Across Parkinson’s (ASAP) Collaborative Research Network, Chevy Chase, MD USA; 4 University of Minnesota Medical School, 420 Delaware Street SE, Minneapolis, MN 55455 USA

**Keywords:** α-Synuclein, Tau, Parkinson’s disease, Lewy body disease, Neurodegeneration

## Abstract

**Background:**

Neuronal dysfunction and degeneration linked to α-synuclein (αS) pathology is thought to be responsible for the progressive nature of Parkinson’s disease and related dementia with Lewy bodies. Studies have indicated bidirectional pathological relationships between αS pathology and tau abnormalities. We recently showed that A53T mutant human αS (HuαS) can cause post-synaptic and cognitive deficits that require microtubule-associated protein tau expression. However, the role of tau in the development of αS pathology and subsequent neuronal dysfunction has been controversial. Herein, we set out to determine the role of tau in the onset and progression of αS pathology (α-synucleinopathy) using a transgenic mouse model of α-synucleinopathy lacking mouse tau expression.

**Methods:**

Transgenic mice expressing A53T mutant HuαS (TgA53T) were crossed with mTau^−/−^ mice to generate TgA53T/mTau^−/−^. To achieve more uniform induction of α-synucleinopathy in mice, we used intramuscular injections of αS preformed fibrils (PFF) in non-transgenic (nTg), TgA53T, TgA53T/mTau^−/−^, and mTau^−/−^ mice. Motor behavior was analyzed at 70 days post inoculation (dpi) of PFF and tissues for biochemical and neuropathological analysis were collected at 40 dpi, 70 dpi, and end stage.

**Results:**

Loss of tau expression significantly delayed the onset of motor deficits in the TgA53T model and the progression of α-synucleinopathy disease, as evidenced by a significant reduction in histopathological and behavioral markers of neurodegeneration and disease, and a significant improvement in survival. In vitro application of PFF to primary mouse hippocampal neurons demonstrated no changes in PFF uptake and processing or pS129 αS aggregation as a function of tau expression. However, PFF-induced neurotoxicity, including morphological deficits in nTg neurons, was prevented with tau removal.

**Conclusions:**

Collectively, our data suggest that tau is likely acting downstream of αS pathology to affect neuronal homeostasis and survival. This work further supports the investigation of tau in α-synucleinopathies to identify novel disease-modifying therapeutic strategies.

**Supplementary Information:**

The online version contains supplementary material available at 10.1186/s40035-022-00309-x.

## Background

Parkinson’s disease (PD) and related dementia with Lewy bodies are progressive neurodegenerative diseases, collectively termed α-synucleinopathies, characterized by degeneration of multiple neuronal populations, particularly the dopaminergic neurons of the substantia nigra pars compacta (SNpc). Another disease hallmark is the presence of intracellular proteinaceous inclusions called Lewy bodies (LBs) and Lewy neurites (LNs). While the SNpc dopaminergic neurodegeneration is responsible for the parkinsonian motor symptoms, the neuropathology and clinical abnormalities in PD extend beyond the SNpc dopaminergic neurons [1, 2], leading to non-motor symptoms, including cognitive dysfunction. The presynaptic protein α-synuclein (αS) is established as a pathogenic-capable protein and genetic mutations in the αS gene (*SNCA)* are causal for autosomal-dominant familial PD. Additionally, mutant αS causes neurodegeneration in cellular and in vivo models, and lastly, αS is the major structural component of LBs and LNs [3], collectively suggesting αS as a central player in disease pathophysiology.

Previously, we showed that mutant αS can cause synaptic and memory deficits that require the expression of microtubule-associated protein tau [4, 5]. While our studies showed that some of the mutant αS-dependent motor deficits are not tau-dependent, other studies showed that αS pathology and the resulting neuronal dysfunction may be mediated by tau. Specifically, while the role of tau in the αS pathology cascade has shown mixed results [6–9], a recent study correlates αS pathology with increased tau oligomers, and shows that neutralization of tau oligomers via systemic injection of a tau oligomer-specific antibody reduces αS aggregation and behavioral deficits in an A53T mutant human αS transgenic mouse model (TgA53T, line M83 [10]). However, a series of more recent studies demonstrates that tau does not impact the onset and transmission of αS pathology in vivo [6, 11]. To address these discrepancies and test for a pathological link between αS and tau, we tested whether the loss of tau expression impacts the onset and progression of overexpressed αS in our TgA53T mouse model of α-synucleinopathy. In this study, we set out to determine the effects of tau on α-synucleinopathy by intramuscular injections of αS preformed fibrils (PFF) [12] to TgA53T mice [3] that are either on wild-type mouse tau or mTau^−/−^ (endogenous mouse tau knockout) background.

## Materials and methods

### Animals

The TgA53T mice were hemizygous for a transgene expressing the human mutant A53T α-synuclein controlled by the mouse prion promotor (αS, hαS^A53T^: line G2-3) and maintained in the C57BL/6J background strain (stock 006823, Jackson Labs; Bar Harbor, ME) [3]. Non-transgenic (nTg) controls came from within these litters. To generate transgenic animals expressing hαS^A53T^ (TgA53T) and lacking endogenous mouse tau (mTau^−/−^), TgA53T males were successively bred to Mapt^tm1(EGFP)Klt^/J females (stock 004779, Jackson Labs) [13] as previously described [4]. Resulting TgA53T/mTau^−/−^ males were bred with mTau^−/−^ females to produce TgA53T/mTau^−/−^ and mTau^−/−^ control mice. This breeding strategy allowed the full use of random littermate controls for the experiments. Moreover, because all mice used here were in the C57BL/6J background, TgA53T and nTg mice used for controls were separately generated. All experimental groups included mixed sex and sample size is specified within figure legends of each analysis. All animal studies were performed in accordance with the NIH guidelines for the use of animals in research and approved by the Institutional Animal Care and Use Committee at the University of Minnesota.

### Antibodies

Primary antibodies used for immunohistochemistry and immunoblots are listed in Additional file 1: Table S1.

### Mouse perfusion and sample collection

Mice were anaesthetized with isoflurane and transcardially perfused with potassium-free phosphate-buffered saline (PBS). The brain was extracted and one hemisphere was placed in 4% paraformaldehyde (PFA) fixation solution for histological processing, while the other hemisphere was sub-dissected for biochemical analysis [4, 14–16].

### Immunohistochemical analysis

After perfusion and dissection, brains and spinal cords were post-fixed in 4% PFA and embedded in paraffin. Sections were cut at 7 μm and immunostained using an immunoperoxidase method with diaminobenzidine as previously described [15–17]. Images were captured using a Leica DM2500 and percent area was quantified within the grey area of the lumbar spinal cord using StereoInvestigator (MBF Biosciences, Williston, VT) and ImageJ/Fiji (NIH). Motor neurons were quantified as previously described [16]. Briefly, using hematoxylin-stained lumbar sections, criteria for motor neurons in the ventral horn included round, open, pale cross-sectioned nucleus (not condensed and darkly stained), and soma diameter of ~ 30 to 40 μm.

### PFF preparation

Human wild-type (HuWT) αS recombinant protein was isolated and purified following the protocol established by Volpicelli-Daley et al. [18]. Purified monomeric αS protein was added to PBS to a concentration of 5 mg/ml and placed at 37 °C with continuous shaking for 5–7 days using a ThermoMixer (Eppendorf; Hamburg, Germany). Assembly of amyloid αS fibrils was assessed twice daily using Thioflavin T fluorometry. Upon Thioflavin T saturation, αS PFF was aliquoted and stored at − 80 °C for future use. Subsequently, a sedimentation assay was performed to confirm a pelletable fraction at 100,000*g* (30 PSI) using a Beckman Airfuge CLS air-driven ultracentrifuge (Beckman Coulter; Indianapolis, IN). The resulting pellet was resuspended in PBS and run alongside the supernatant using SDS-PAGE (BioRad) and Coomassie Brilliant Blue (Thermo Fisher Scientific) staining. HuWT αS PFF was prepared for use from 5 mg/ml frozen stock diluted with PBS to a concentration of 0.25 mg/ml. The fibrils were subsequently sonicated utilizing a Fisher Scientific Branson micro probe tip sonicator (Fischer Scientific; Hampton, NH) 60 pulses, 1 s ON, 1 s OFF for 120 s, at 20% amplitude immediately prior to use. In addition, the endotoxin unit (EU) levels were assayed in the PFF preparations using a Pierce Chromogenic Endotoxin Quant Kit (ThermoFisher Scientific) and determined to be < 0.1 EU/ml.

### Intramuscular injections

**Fig. 1:**
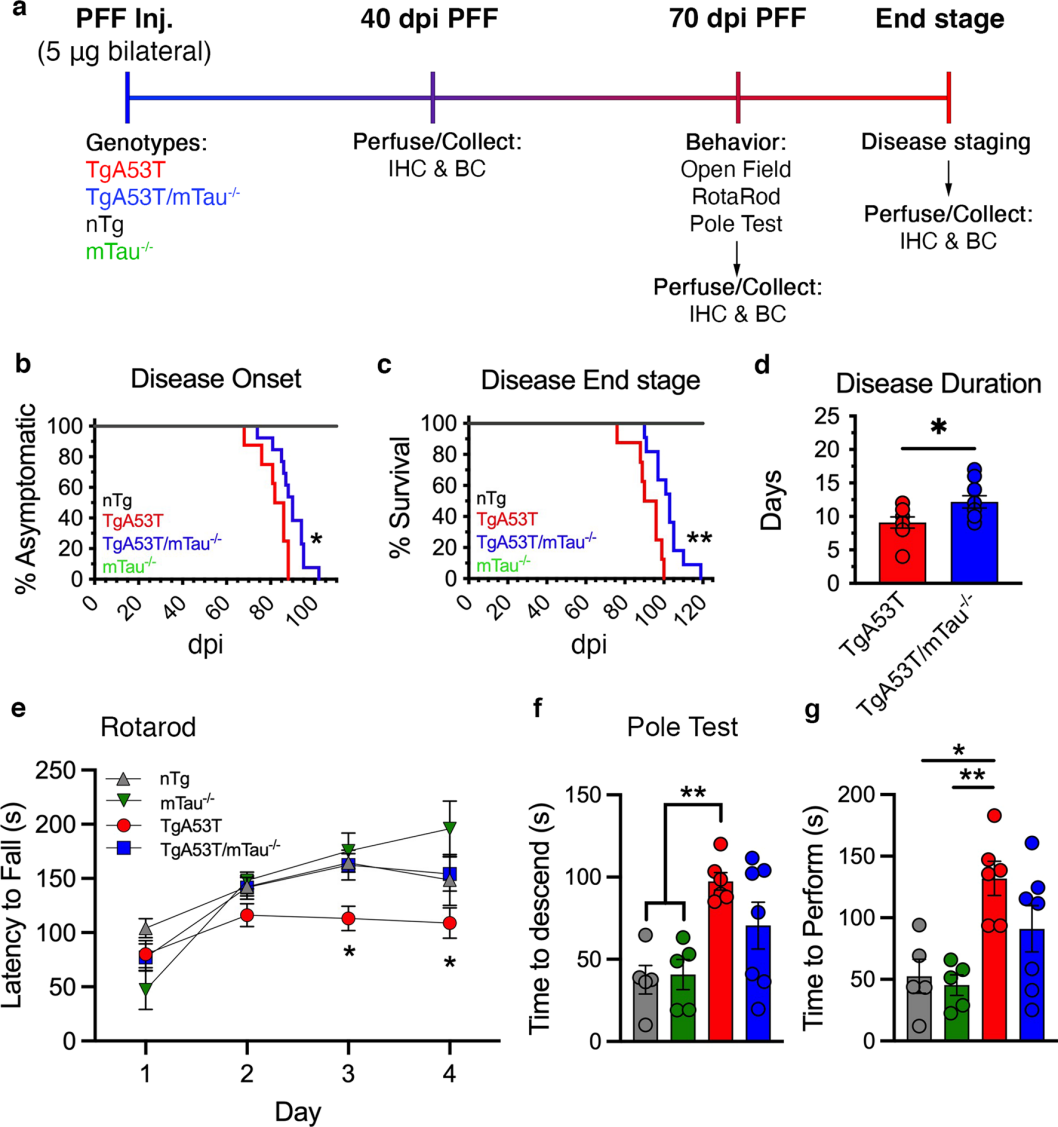
Loss of tau in TgA53T mice delays disease onset and progression following αS PFF inoculation. **a** Schematic of study paradigm, including genotypic groups, collection time points, behavior, and disease staging analysis. **b**, **c** Kaplan–Meier plots of all groups showing disease onset (onset of motor abnormalities) (**b**; *P* = 0.0122; Log-rank (Mantel Cox) test) and time to reach end stage (hind limb paralysis) (**c**; *P* = 0.0022; Log-rank (Mantel Cox) test). Both onset and time to end stage were delayed in TgA53T mice lacking tau (TgA53T/mTau^−/−^). **d** Average duration from disease onset to end stage was also significantly extended with loss of tau (*P* = 0.0247; *t*-test). **e** Performance on rotarod at 70 dpi showed that TgA53T mice exhibited decreased latency to fall compared to TgA53T/mTau^−/−^ subjects. One-way ANOVA with Tukey’s post-hoc analysis; Day 3: *F*_(3,18)_ = 4.925, *P* = 0.0114; Day 4: *F*_(3,18)_ = 3.150, *P* = 0.0505. **f**, **g** TgA53T mice required more time to descend the pole (**f**) and complete (turn and descend) the pole test (**g**) compared to nTg and mTau^−/−^ controls, while performance of the TgA53T/mTau^−/−^ mice was not significantly different from all other groups, indicating that loss of tau expression results in partial protection from impaired performance. Abbreviations: preformed fibril, PFF; days post inoculation, dpi; immunohistochemistry, IHC; biochemistry, BC; seconds, s. One-way ANOVA with Tukey’s post-hoc analysis; time to descend: *F*_(3,20)_ = 4.186, *P* = 0.0188; time to perform: *F*_(3,20)_ = 4.220; *P* = 0.0182. *n* = 8–11 animals/genotype (survival), *n* = 5**–**7 animals/genotype (behavior). *P < 0.05 and **P < 0.01; error bars represent mean ± SEM

Intramuscular injections of PFF were performed as previously described [12]. In the TgA53T mouse model, IM PFF injection leads to the pattern of α-synucleinopathy similar to that seen with aging but occurs with more predictable and uniform disease development. In our lab, the current average life-span of TgA53T mice is 12.5 ± 1.5 months (mean, SEM) with ~ 15% of mice escaping disease at 2 years of age. The life-span of IM PFF-injected TgA53T mice is 91.75 ± 2.8 days (mean, SEM) with 100% of mice developing disease (Fig. 1). Because the disease progression is highly predictable following PFF inoculation, presymptomatic analysis is possible without greatly increasing the number of mice examined. Each mouse was first anesthetized using an isoflurane chamber until unresponsive to stimuli. Immediately after that, 5 µg of PFF was injected into the *bicep femoralis* muscle bilaterally. The mouse was then allowed to recover on a heat pad and was placed back to its home cage. Following PFF injections, the mice were evaluated for disease onset and progression three days per week (Monday, Wednesday, Friday) starting at 60 days post PFF injection. Disease onset was identified by an imbalance in gait leading to a wobbling phenotype. This progresses to hindlimb slipping and a failure to properly swing the hindfoot forward. Finally, the end stage was characterized as complete hindlimb paralysis. Mice were injected between 3 and 5 months of age and those that did not develop disease onset in the end stage cohort following PFF inoculation (i.e. nTg and mTau^−/−^ genotypes) were collected at 120 days post inoculation. The end-stage cohort also included TgA53T mice injected with vehicle (saline), of which none developed disease prior to collection along with the PFF-injected mice.

### Motor behavior tests

Rotarod and open-field tests were performed as previously described [4]. Briefly, mice were subjected to the rotarod test for a total of four trials per day (with a minimum interval of 20 min between trials), for four days with a maximum trial length of 5 min, with rod accelerating from 5 to 50 rpm. For the pole test [19], mice were placed on a horizontal pole facing the top. The pole was then placed upright with the mouse facing upward. The time to turn, time to descend to the bottom of the pole, and cumulative time to perform (both time to re-orient/turn and descend) the pole test were recorded with a maximum duration of 120 s. All animals were tested for 3 trials with 20 min of rest in-between trials. All behavioral tests were performed between 60 to 70 days post PFF inoculation.

### Western blot (immunoblot) analysis

Brain regions and spinal cord were dissected out and stored at − 80 °C until homogenization as described [4, 14, 20–23]. SDS-PAGE separation of proteins in brain lysates and transfer of resolved proteins to nitrocellulose membranes were performed as previously described [4, 14, 20]. Briefly, frozen tissues were homogenized in TNE buffer consisting of TNE solution: Tris–HCl 50 mM, NaCl 150 mM and EDTA 5 mM, and HALT protease and phosphatase inhibitors (Thermo-Fisher; Waltham, MA). For total lysate preparation, samples were then supplemented with 0.5% NP40, 0.5% DOC, and 1% SDS. For TX-100 fractionation, TNE homogenates were added to 1% TX-100 and sonicated prior to centrifugation at 16,000*g* for 20 min. Supernatant was collected as the soluble fraction while the pellet was washed and centrifuged again in 1% TX-100. The resulting pellet was then reconstituted in TNE with detergents as the TX-100 insoluble fraction. Homogenates were then sonicated and heated at 100 °C for 10 min and centrifuged for 10 min at 16,000*g*. The supernatant was collected and BCA assay (Pierce, Thermo; Rockford, IL) was performed. Samples were prepared to equal protein concentrations in reducing, SDS-sample, Laemmli buffer (Boston BioProducts; Ashland, MA). For Western blot analysis, protein lysates were run on Criterion™ TGX™ gels (BioRad, Hercules, CA) and transferred onto nitrocellulose membranes. Proteins on membranes were detected using appropriate primary antibodies followed by horseradish peroxidase **(**HRP)-conjugated secondary antibodies (Invitrogen, Carlsbad, CA). Membranes were then developed using chemiluminescent substrates (BioRad and Thermo) and the ImageQuant LAS 4000 detection system (GE Life Sciences, Piscataway, NJ). Densitometry on Western blot images was analyzed using the ImageQuant TL 8.1 software (GE Life Sciences).

### Dot blot analysis

Dot blot analysis for proteins and oligomeric structures was performed as previously described [14, 24]. Briefly, samples were prepared first by adding 10 µl of 1× TNE buffer (consisting of Tris–HCl, 50 mM; NaCl, 150 mM; and EDTA, 5 mM) with protease and phosphatase inhibitors per 1 mg of tissue and mechanically homogenized. Samples were then centrifuged at 100,000*g* for 10 min. The supernatant was then collected and immunodepleted using Protein A and Protein G Mag Sepharose Xtra beads (MilliporeSigma; St. Louis, MO). The pellet was reconstituted in 1× TNE + 1% TritonX-100 (TX-100) with protease and phosphatase inhibitors, sonicated (10 s ON, 2 s OFF, 20% amplitude) for 2 cycles, then centrifuged for 20 min at 16,000*g* at 4 °C and supernatant separated. The TX-100-insoluble fraction was resuspended in 1× TNE with protease and phosphatase inhibitors. The samples were then diluted to 1 µg/µl (0.5 µg/µl for insoluble) and 2.5 µl droplets were deposited onto a nitrocellulose membrane and allowed to dry for 30 min. The membrane was activated in transfer buffer with 10% methanol, microwaved in 50 ml PBS for 25 s, let sit for 3 min, followed by another 15 s in the microwave. The membrane was allowed to cool down prior to blocking with tris-buffered saline containing 5% bovine serum albumin for 30 min immediately prior to overnight incubation with the primary antibody at 4 °C. The membranes were then washed and incubated for 1 h with respective IRDye secondaries and imaged using the Li-Cor Odyssey CLx system (LiCor Biosciences; Lincoln, NE).

### Primary neuron dissection and culture

Mouse pups between postnatal days 0–2 (P0–2) were used for the isolation of primary hippocampal neurons. The hippocampus was removed and placed in cold Hibernate-A medium (BrainBits LLC; Springfield, IL). Hippocampi were then transferred to a digestion medium containing Hibernate-A-CaCl_2_ (BrainBits LCC), papain, *L*-Cysteine, and EDTA for 15 min at 37 °C with occasional gentle shaking. The hippocampi were gently washed in a digestion inhibitor solution and subsequently triturated. The cells were plated in a plating medium at either a density of 650,000 or 250,000 cells per well in a 12-well culture dish (for protein and immunocytochemical analysis, respectively) and the medium was replaced with NbActiv4 (BrainBits LCC) + FdU mitotic inhibitor the following day. PFF was added to the primary cultures between 7 and 10 days in vitro (DIV) at a concentration of 1 μg/ml for immunocytochemical analysis, and 4 μg/ml for western blot analysis. For uptake analysis, PFFs were removed following 2 h of incubation, the cells washed in fresh medium, then replaced with fresh medium. The uptake time points started at time 0 h when PFF was washed from the culture (i.e., 2 h post treatment). For immunocytochemical analysis, 1 μg/ml of PFF was added and the neurons were allowed to continue in culture for up to 14 days.

### Immunocytochemistry

Cells were briefly washed with cold PBS followed by addition of cold 4% PFA and incubated at room temperature for 30 min. The cells were then rinsed with PBS five times for 5 min per wash and then blocked with 50% Sniper (Biocare Medical; Pacheco, CA) for 15 min at room temperature (RT). The cells were then rinsed with PBS twice for 5 min followed by incubation with the primary antibody in 5% Sniper, 0.1% TritonX-100 in PBS overnight at 4 °C. Cells were then rinsed three times for 5 min followed by incubation with the secondary antibody in 5% Sniper, 0.1% TX-100 in PBS for 2 h at RT. Cells were incubated with DAPI prior to cover slipping in Antifade Vectashield (Vector Laboratories; Burlingame, CA). Quantification of imaged stains utilized threshold-based area measurements using ImageJ/Fiji software (NIH). In some cases, neuronal nuclei were counted manually, and area-based quantification was normalized to either cell number, or percent dendritic area.

### Statistical analysis

All statistical analyses were performed in Prism 9.0.2 (GraphPad Software; San Diego, CA). Results are expressed as mean ± SEM (standard error of the mean). Student’s *t*-test was used for comparison of only two groups. Kaplan–Meier plots were evaluated using the Log-rank Mantel Cox test. Where applicable, normality was tested using either D’Agostino–Pearson omnibus or Shapiro–Wilk tests prior to choosing the appropriate statistical test. When comparing one variable across more than two groups, one-way ANOVA was performed with Tukey’s post-hoc analysis for multiple comparisons. Two-way ANOVA was performed when comparing two variables on multiple groups with Sidak’s post-test for multiple comparisons. Statistical significance was set for α = 0.05. Data representations are described in figure legends.

## Results

### Loss of tau delays the onset of motor symptoms after PFF inoculation

We previously showed that tau is required for synaptic and memory deficits caused by A53T mutant human α-synuclein (HuαS^A53T^) expression in primary neurons and in the TgA53T model [4, 5]. While tau expression did not impact αS-dependent motor abnormalities in presymptomatic mice, studies have proposed that a pathological relationship exists between tau and αS. Interestingly, recent studies show that while αS promotes pathological spreading of tau in brain [6], tau did not promote spreading of αS pathology [6, 11]. Therefore, we aimed to further evaluate the role of tau in the onset and progression of α-synucleinopathy derived from HuαS.

In order to induce αS pathology in a temporally regulated manner, we used intramuscular injections of wild-type (WT) HuαS PFF into our TgA53T mouse model [12], as well as TgA53T/mTau^−/−^ and genotypic controls (mTau^−/−^ and nTg; Fig. 1a). Following PFF inoculations, tissue samples for histology and biochemical analyses were collected at 40 and 70 days post inoculation (dpi), as well as when the mice reached the disease end stage (classified as ataxic deterioration to the point of complete hindlimb paralysis preventing ambulation; Fig. 1a). In addition, prior to 70 dpi, mice underwent a battery of behavioral tests: pole, rotarod, and open field testing.

All PFF-inoculated TgA53T animals in the wild-type background developed motor symptoms and reached end stage by ~ 100 dpi. Significantly, the average times to reach ataxic onset and end stage were significantly delayed in TgA53T/mTau^−/−^ compared to TgA53T (*P* = 0.0122 and *P* = 0.0022, respectively; Fig. 1b, c). Moreover, the progression from initial onset of motor symptoms to end stage was also significantly prolonged in TgA53T/mTau^−/−^ compared to TgA53T (*P* = 0.0247; Fig. 1d), indicating that the loss of tau delayed the progression of α-synucleinopathy-associated neurodegeneration.

Consistent with the delay in disease progression associated with the loss of endogenous mTau expression, behavioral analysis at 70 dpi also showed that loss of tau significantly attenuated HuαS^A53T^-mediated behavioral abnormalities in TgA53T mice. The rotarod test of motor coordination showed that while TgA53T mice had a significantly decreased latency to fall compared to all other groups on day 3 and to mTau^−/−^ controls on day 4, the TgA53T/mTau^−/−^ mice had a similar latency to fall as control mice (Fig. 1e). In the pole test, an additional test of motor coordination, the TgA53T mice required significantly more time to both descend the pole, as well as for total performance (cumulative time to re-orient on the pole and descend to the base) than the control mice. The mean times for descending the pole and for total performance of the TgA53T/mTau^−/−^ mice was intermediate compared to TgA53T mice and control mice but was not significantly different from either group (Fig. 1f, g). As previously documented [4], the TgA53T and TgA53T/mTau^−/−^ groups exhibited similar hyperactivity in the open field test (Additional file 1: Fig. S1a). Interestingly, while the TgA53T group presented increased anxiety-associated behavior as they spent less time in the center, such behavior was not observed in the TgA53T/mTau^−/−^ mice (Additional file 1: Fig. S1b, c). Collectively, these results show that the loss of tau expression delays the onset and progression of overt disease as well as ameliorating behavioral deficits in the TgA53T model of α-synucleinopathy.

### Loss of tau expression does not impact phospho-serine129 αS (pS129 αS) pathology in  end-stage TgA53T mice but leads to modest reduction in pS129 αS accumulation in presymptomatic TgA53T mice

The delay in disease/ataxic onset, as well as behavioral deficits, in the TgA53T/mTau^−/−^ mice compared with TgA53T mice seems to contradict prior studies using the mouse αS PFF inoculation model [6, 11]. Thus, we asked whether lack of tau expression impacts subcortical α-synucleinopathy in the TgA53T model.

**Fig. 2:**
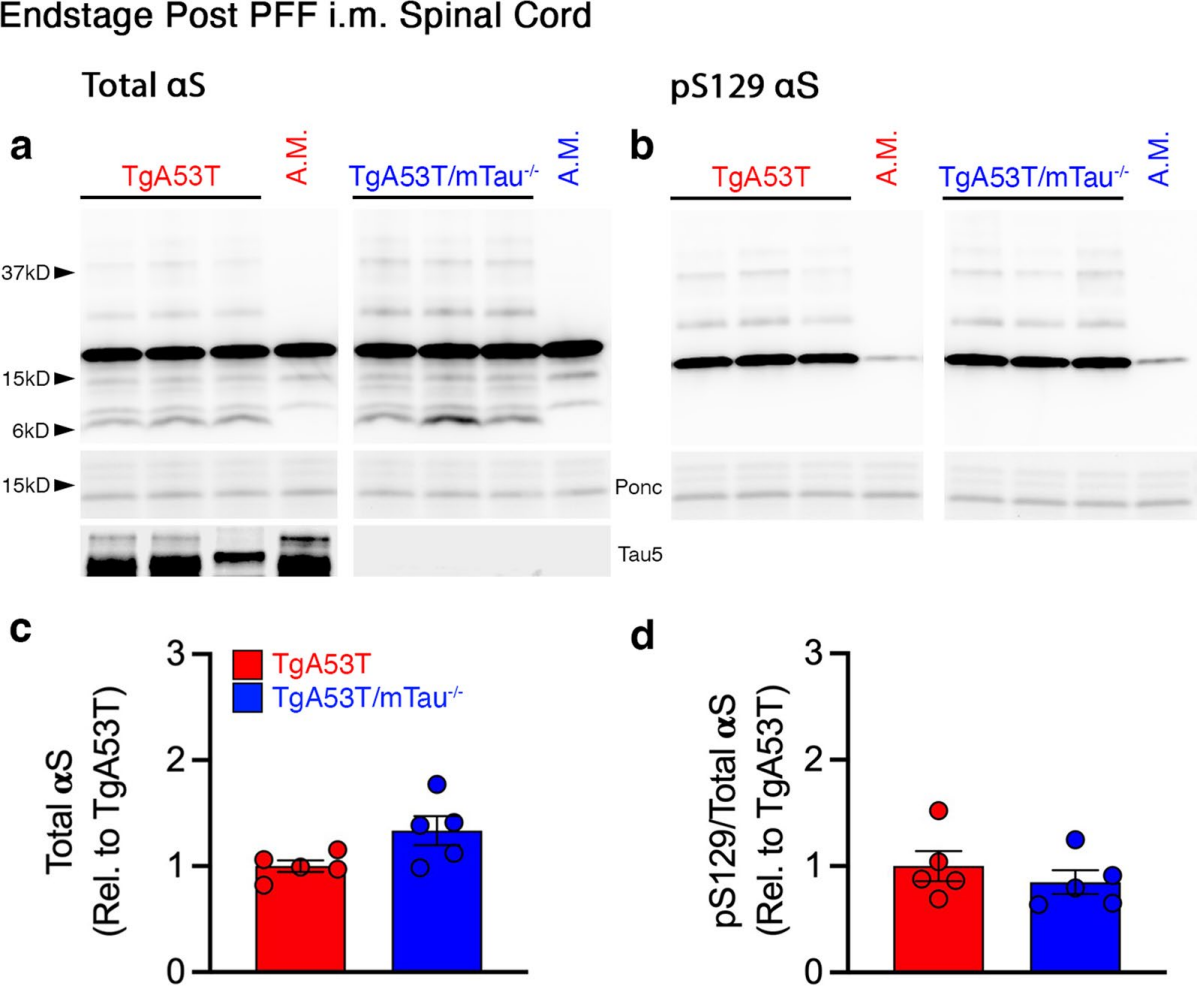
Biochemical abundance of pS129 αS in spinal cord is not affected by tau expression in end-stage TgA53T mice. **a** Representative immunoblots of total αS (**a**) and pS129 αS (**b**) in spinal cords of end-stage mice. **c**, **d** Quantitation of immunoblots shown in **a** and **b**. The level of pS129 αS was normalized to total αS. The levels of total αS and pS129 αS/total αS were not different between TgA53T and TgA53T/mTau^−/−^ mice in end-stage spinal cord lysates (*t*-test: *P* = 0.0518 and *P* = 0.4267, respectively). All quantified bands were normalized to the respective ponceau S total protein. Abbreviations: age-matched, A.M.; arbitrary units, A.U.; ponceau S, ponc; *n* = 5 animals/genotype; error bars represent mean ± SEM

To survey if the expression of tau was correlated with bulk changes in αS pathology, we performed biochemical analysis of lysates from spinal cord and brain stem regions that are prone to develop αS pathology in the TgA53T model [3, 15]. Immunoblot analysis of spinal cord from end-stage mice showed similar levels of full-length αS compared to age-matched controls (Fig. 2a) and a prominent increase in pS129 αS, a marker of pathological αS, in both TgA53T and TgA53T/mTau^−/−^ mice (Fig. 2b**)**. Further, consistent with a prior study [6], no difference in the levels of pS129 αS/total αS was observed between TgA53T and TgA53T/mTau^−/−^ mice (Fig. 2c, d). To determine if there was a difference in the aggregation of αS that is not fully reflected by total pS129 αS levels, we examined Triton X-100 detergent-soluble and -insoluble fractions from the spinal cord (Additional file 1: Fig. S2). As expected from prior studies [4, 20], most of the pS129 αS was present in the detergent-insoluble fraction while the detergent-soluble fraction contained very little pS129 αS (Additional file 1: Fig. S2a). Our results showed that while PFF inoculation increased the amount of insoluble αS, expression of tau did not significantly impact the levels of insoluble αS, but there was slightly increased αS in the soluble fraction (Additional file 1: Fig. S2). Moreover, tau expression did not impact the levels of SDS-stable oligomers that resolve at ~ 25 kDa, ~ 37 kDa, and > 200 kDa (Additional file 1: Fig. S2d, g). Analysis of the brainstem region showed that PFF-inoculation led to similar increases in pS129 αS levels in both TgA53T and TgA53T/mTau^−/−^ mice at the end stage (Additional file 1: Fig. S3a, b), similar to the patterns observed in the spinal cord.

**Fig. 3:**
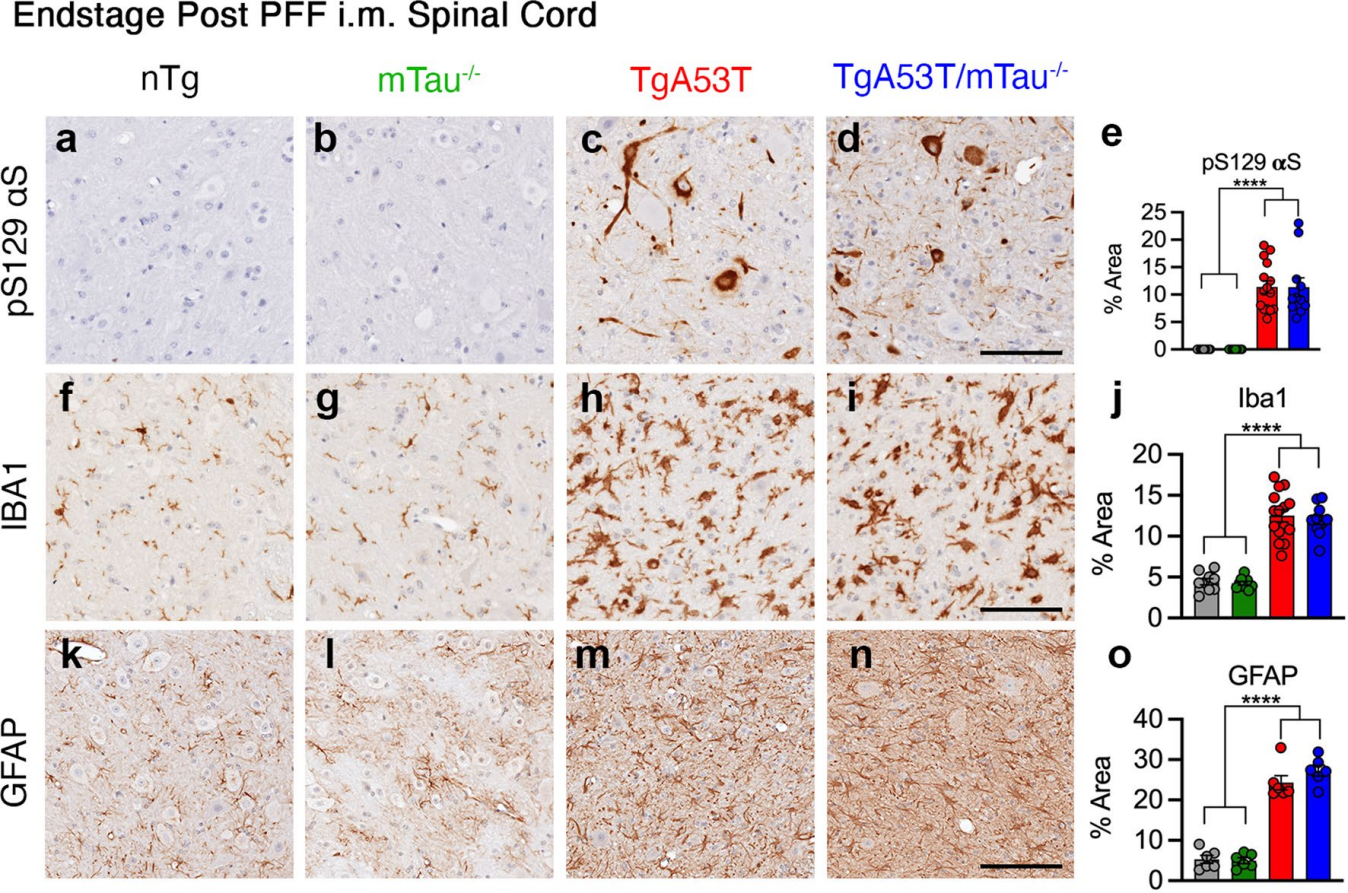
Loss of tau does not affect end-stage neuropathology in TgA53T mice. Spinal cord sections from end-stage nTg, mTau^−/−^, TgA53T and TgA53T/mTau^−/−^ mice were stained for pS129 αS (**a–e**), Iba1 (microglia) (**f–j**), and GFAP (astrocytes) (**k–o**). Shown are representative images and corresponding quantitative analysis of % area covered by immunoreactivity in the lumbar region. In both TgA53T and TgA53T/mTau^−/−^ mice the indices of neuropathology were significantly increased to a similar extent. One-way ANOVA with Tukey’s post-hoc analysis. pS129 αS: *F*_(3,40)_ = 30.64; *P* < 0.0001. Iba1: *F*_(3,40)_ = 54.81; *P* < 0.0001. GFAP: *F*_(3,20)_ = 88.16; *P* < 0.0001. *n* = 6–15 sections from 3 to 5 animals/genotype; scale bars = 100 μm; error bars represent mean ± SEM

We performed histological analysis for αS pathology (pS129 αS) to determine if the pattern of αS pathology was influenced or mediated by tau expression. The immunostained lumbar spinal cord sections were used to determine the percent (%) area covered by immunoreactivity. Consistent with the bulk biochemical analysis, our results showed that both TgA53T and TgA53T/mTau^−/−^ mice developed similar levels of pS129 αS pathology at end stage while the control animals (nTg and mTau^−/−^ alone) did not exhibit pS129 αS staining (Fig. 3a–e). In addition, we also examined activation of microglia and astrocytes by Iba1 and GFAP immunostaining, respectively. In end-stage animals, quantitative analysis of microglial activation (Iba1) showed significant activation with αS pathology but did not reveal any differences as a function of tau expression (Fig. 3f–j). Similarly, while the increase in GFAP staining occurred with αS pathology, there were no differences as a function of tau expression (Fig. 3k–o; see Additional file 1: Fig. S4 for representative low-magnification images). Importantly, pS129 αS histopathology and neuroinflammation (i.e., increased Iba1 and GFAP abundance) were only observed after injection of αS PFF to TgA53T overexpression mice but not in age-matched TgA53T without αS PFF injection, or age-matched nTg mice with or without αS PFF injection (Additional file 1: Fig. S5). In addition, pS129 αS, Iba1, and GFAP reactivity were elevated in end-stage brainstem, cerebellum, and motor cortex regions, without any obvious qualitative differences between TgA53T and TgA53T/mTau^−/−^ mice (Additional file 1: Fig. S6).

**Fig. 4:**
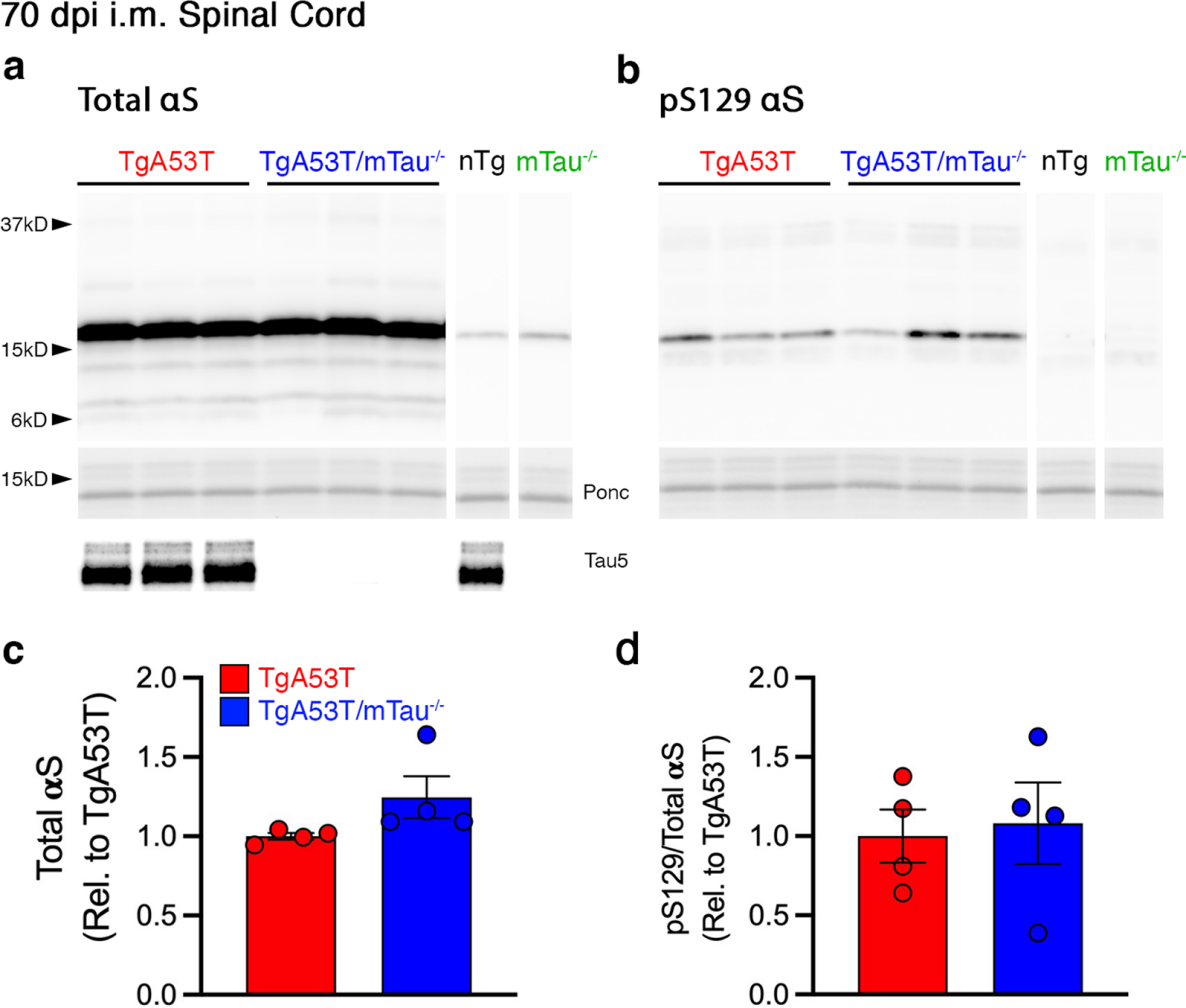
Bulk tissue level of pS129 αS in spinal cord is not affected by tau expression in TgA53T mice at 70 dpi. **a** Representative immunoblots of total αS (**a**) and pS129 αS (**b**) in spinal cord of 70-dpi mice. **c**, **d** Quantitation of immunoblots shown in **a** and **b**. Total αS (**c**), and pS129 αS normalized to total αS (**d**) were not different between TgA53T and TgA53T/mTau^−/−^ mice in 70-dpi spinal cord lysates (*t* test, *P* = 0.1575 and *P* = 0.7993, respectively). All quantified bands were normalized to the respective ponceau S total protein. Abbreviations: ponceau S, ponc; arbitrary units, A.U.; *n* = 4 animals/genotype; error bars represent mean ± SEM

Because end stage of disease is reached at a later dpi in TgA53T/mTau^−/−^ mice, it is possible that αS pathology was normalized between groups at terminal stages. Thus, we evaluated tissues at the same presymptomatic stage following PFF inoculation (70 dpi and 40 dpi). Biochemical analysis showed that at 70 dpi, the relative levels of pS129 in the spinal cord were increased over nTg mice but at levels much lower than at the end stage. Further, the immunoblots of 70 dpi spinal cord (Fig. 4b) and brain stem lysates (Additional file 1: Fig. S3c, d) failed to show any differences in pS129 αS levels or insoluble αS as a function of tau expression (Additional file 1: Fig. S7e, g). Moreover, tau expression did not impact the levels of SDS-stable αS oligomers (Additional file 1: Fig. S7e, f, i, j).

**Fig. 5:**
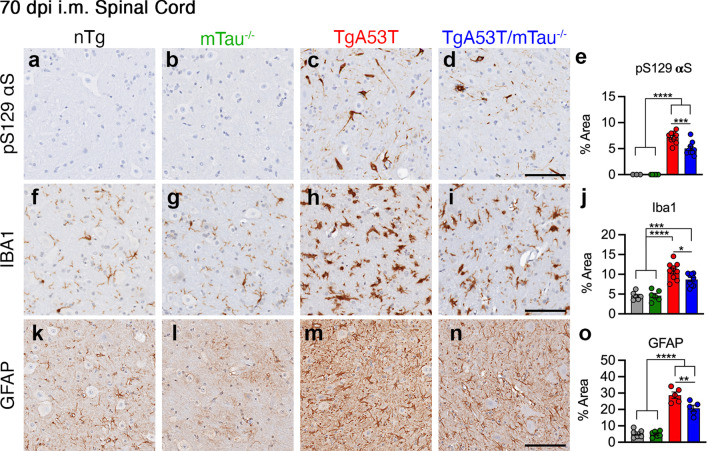
Loss of tau attenuates intermediate neuropathology in TgA53T mice. Spinal cord sections from 70-dpi nTg, mTau^−/−^, TgA53T, and TgA53T/mTau^−/−^ mice were stained for pS129 αS (**a–e**), Iba1 (**f–j**), and GFAP (**k–o**). Shown are representative images and corresponding quantitative analysis of % area covered by immunoreactivity in the lumbar region. The indices of neuropathology were all significantly reduced in TgA53T/mTau^−/−^ compared to TgA53T mice at 70 dpi. One-way ANOVA with Tukey’s post-hoc analysis. pS129 αS: *F*_(3,23)_ = 82.46; *P* < 0.0001. Iba1: *F*_(3,26)_ = 25.79; *P* < 0.0001. GFAP: *F*_(3,18)_ = 69.86; *P* < 0.0001. *n* = 5–9 sections from 3 animals/genotype; scale bars = 100 μm; error bars represent mean ± SEM

We also performed histological analysis in 70 dpi mice for pS129 αS and neuroinflammation as was done for the end-stage subjects above. Significantly, despite the lack of differences in bulk immunoblot analysis of total pS129 αS levels (Fig. 4), quantitative analysis of the pS129 αS staining in the lumbar spinal cord revealed a modest decrease in pS129 αS pathology in TgA53T/mTau^−/−^ compared to TgA53T mice (Fig. 5a-e). This suggested that while the overall abundance of pS129 αS along the spinal cord, measured biochemically (Fig. 4), was unchanged, there was a specific delay in the neuronal accumulation of pS129 αS in the grey matter of the spinal cord.

The analysis of microglial (Fig. 5f–j) and astrocytic (Fig. 5k–o) activation in presymptomatic animals at 70 dpi showed a significant increase in microglial activation in mice with αS pathology. Consistent with the reduced αS pathology in TgA53T/mTau^−/−^ animals, the activation status of microglia and astrocytes was reduced in these mice compared to TgA53T mice (Fig. 5j, o; see Additional file 1: Fig. S8 for representative low-magnification images). In the brainstem, pS129 αS, Iba1, and GFAP were modestly increased at 70 dpi in TgA53T and TgA53T/mTau^−/−^ mice. The overall abundance was qualitatively similar to the quantitative results seen with spinal cord sections (Additional file 1: Fig. S9).

Given that loss of tau expression is associated with reduced αS pathology at 70 dpi, we analyzed animals at 40 dpi to determine if tau expression affects the onset of αS pathology following intramuscular injection. A previous study showed that following intramuscular injection, αS pathology appears in spinal cord between 30 and 60 dpi [25]. Western blot analysis of 40 dpi mice showed no obvious increase in the levels of pS129 αS (Additional file 1: Fig. S10a, b). However, immunohistological analysis of 40 dpi mice showed that sparse pS129 αS pathology could be detected, confirming that the αS pathology was initiated between 30–60 dpi. Quantitative analysis showed that the amount of pS129 αS pathology at 40 dpi was not altered by tau loss (Additional file 1: Fig. S10c–f). Finally, analysis of neuroinflammation (Iba-1 and GFAP) showed that the low level of early αS pathology at 40 dpi was not associated with increased neuroinflammation (Additional file 1: Fig. S10g–n).

### Loss of tau expression leads to reduced neurodegeneration in presymptomatic TgA53T mice but not in end-stage mice

Thus far, our results indicate that while tau is associated with modest increases in αS pathology at 70 dpi, tau seems not to impact the onset of αS pathology at 40 dpi or the extent of αS pathology at end stage. However, it is possible that tau might be acting downstream of αS abnormalities [4, 5]. Because loss of motor neurons is a robust neurodegenerative phenotype in the TgA53T model [16], including the intramuscular injection model [25], we examined if tau expression affects the loss of motor neurons in the spinal cord of TgA53T mice following the PFF injection.

**Fig. 6:**
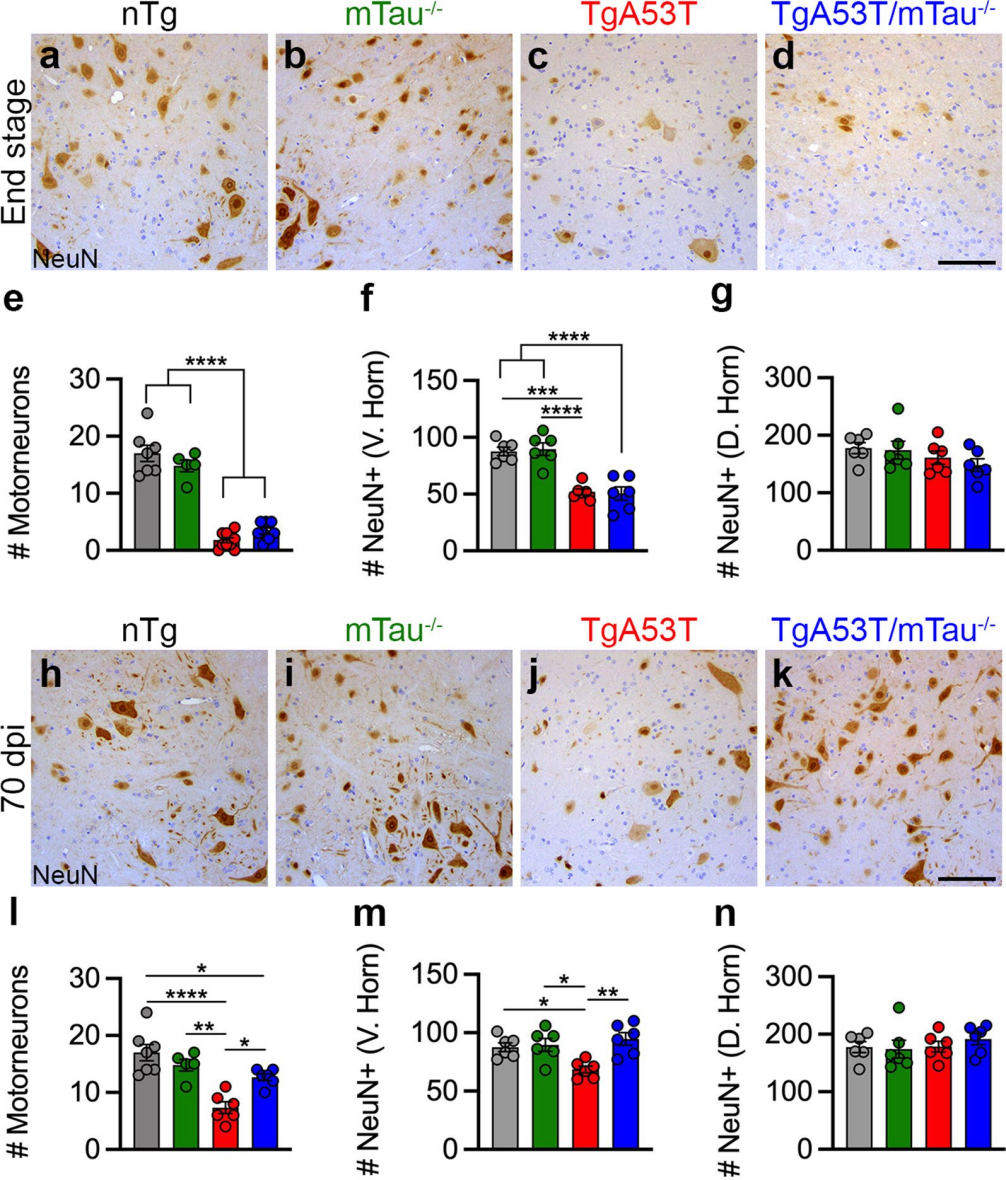
Loss of tau delays α-synucleinopathy-associated spinal motor neuron degeneration in TgA53T mice. Spinal cord sections from  end-stage (**a–g**) and 70-dpi (**h–n**) TgA53T mice and controls (nTg, mTau^−/−^) were stained for NeuN (neuronal marker). Shown are representative images of lumbar ventral horn region. In end-stage mice (**a–g**), TgA53T and TgA53T/mTau^−/−^ mice show significant loss of ventral horn (V. Horn) motor neurons (**a–e**; *F*_(3,26)_ = 82.45, *P* < 0.0001). NeuN quantification further demonstrated significant neurodegeneration within the ventral horn (**f**; *F*_(3,20)_ = 21.75, *P* < 0.0001), but not dorsal horn (D. Horn) (**g**; *F*_(3,20)_ = 1.257; *P* = 0.3160). At 70 dpi (**h–n**), ventral horn motor neuron degeneration (**l**; *F*_(3,20)_ = 14.15, *P* < 0.0001) as well as ventral NeuN+ neuronal loss (**m**—Ventral: *F*_(3,20)_ = 6.490, *P* = 0.0030; **n**—Dorsal: *F*_(3,20)_ = 0.4447, *P* = 0.7237) was delayed with tau loss. Abbreviation: days post inoculation, dpi; One-way ANOVA with Tukey’s post-hoc analysis. *n* = 5–10 sections from 3 to 5 animals/genotype; scale bars = 100 μm; error bars represent mean ± SEM

We analyzed NeuN^+^ neurons in the ventral horn and dorsal horn of both 70-dpi and end-stage mice (see Additional file 1: Fig. S11 for representative low-magnification images and regions analyzed). Our analysis of end-stage mice following PFF inoculation showed that the presence of αS pathology and limb paralysis in the TgA53T mice were accompanied by severe loss of motor neurons in the ventral horn (Fig. 6a–e). Further, the average number of motor neurons per lumbar spinal cord section in TgA53T/mTau^−/−^ mice was not different from TgA53T mice. This was further demonstrated with loss of total NeuN^+^ content of the ventral horn (Fig. 6f), while the dorsal horn neurons were left intact (Fig. 6g). Significantly, analysis of presymptomatic animals at 70 dpi showed that the presence of αS pathology in TgA53T mice was already associated with a significant loss of ventral horn motor neurons in the lumbar spinal cord, albeit less severe than in the end-stage animals (Fig. 6h–l). More importantly, the loss of motor neurons was attenuated in the TgA53T/mTau^−/−^ mice, despite the significant presence of αS pathology, compared to the TgA53T mice (Fig. 6l; see Additional file 1: Fig. S11 for representative low-magnification images). In addition, this protection was also observed after quantification of ventral horn neurons via NeuN^+^ staining (Fig. 6m), while the dorsal horn neurons were not affected (Fig. 6n).

### Loss of tau does not impact soluble α-synuclein oligomer formation or glycogen synthase kinase 3β (GSK3β) activity

Using a different HuαS^A53T^-expressing transgenic mouse line (M83), it has been previously demonstrated that TgA53T (M83) mice accumulate soluble tau oligomers with aging, and treatment with tau-oligomer-specific antibody can subsequently reduce αS oligomers and aggregates [10]. Therefore, while the loss of tau expression does not alter overt αS aggregation or SDS-stable oligomers (Fig. 2, Additional file 1: Fig. S2 and S7), we tested if the loss of tau expression reduces the levels of soluble αS oligomers in our TgA53T model using the Syn33 antibody [4, 10, 24] with non-denaturing dot blot analysis.

**Fig. 7:**
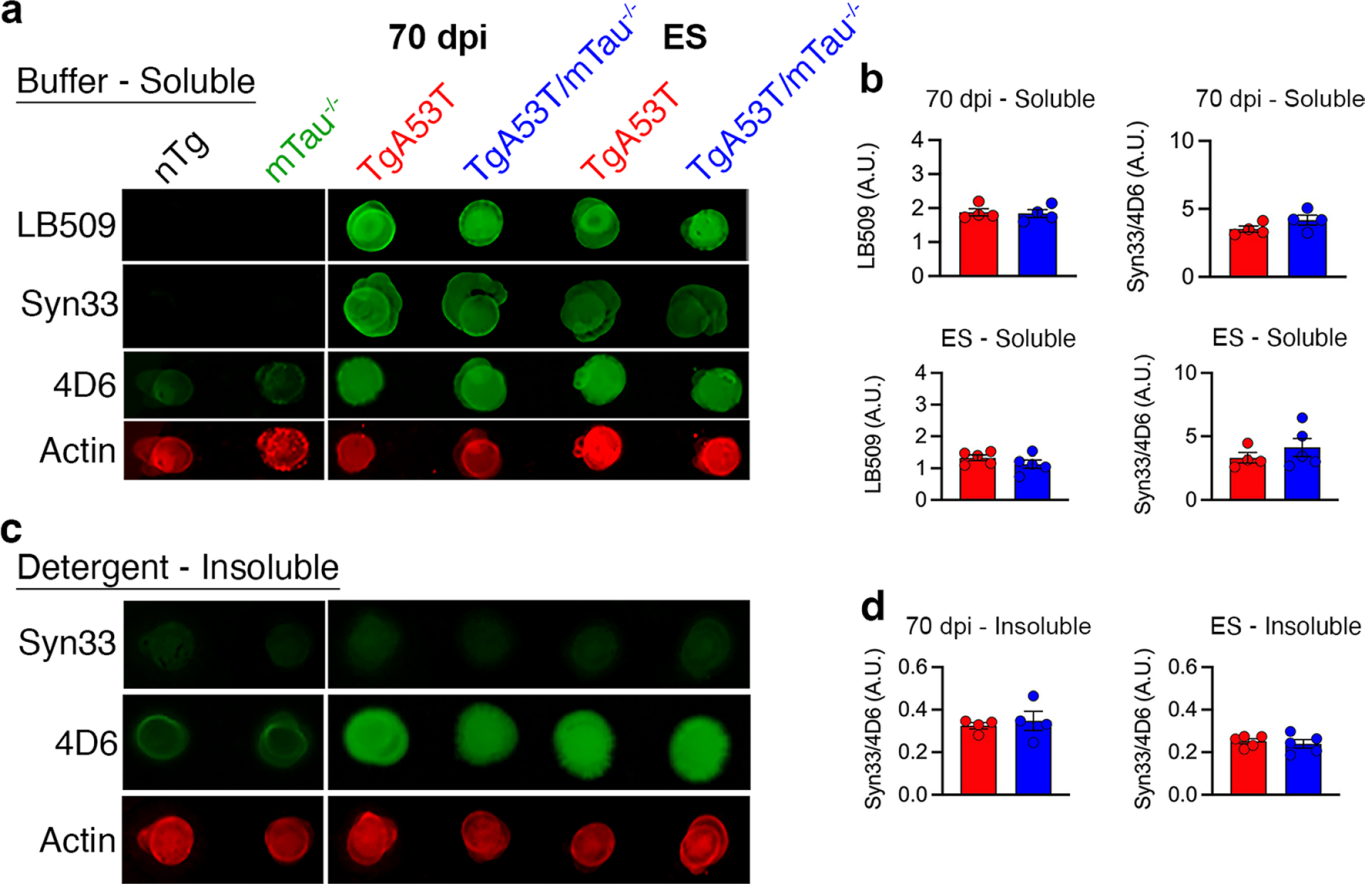
Soluble αS oligomers detected by Syn33 are not affected by tau expression. **a** Dot blot detection of human αS (LB509), αS off-pathway oligomers (Syn33), and total αS (4D6) in buffer-soluble spinal cord fractions of 70-dpi and end-stage mice. **b** No differences in LB509 or Syn33 were observed as a result of tau expression. **c**, **d** Syn33+ αS oligomers were not detected in the detergent-insoluble spinal cord fractions of both 70-dpi and end-stage mice. Abbreviations: days post inoculation, dpi; end stage, ES; arbitrary units, A.U.; *n* = 4–5 animals/genotype; error bars represent mean ± SEM

Dot blot analysis of buffer-soluble fractions from spinal cords of both end-stage and 70-dpi spinal cord lysates from TgA53T animals revealed higher levels of αS oligomers as a function of HuαS^A53T^ expression compared to nTg and mTau^−/−^ controls. Further, the accumulation of Syn33^+^ oligomeric species was comparable between TgA53T and TgA53T/mTau^−/−^ groups (Fig. 7a, b). Consistent with the specificity of Syn33 to soluble oligomers, Syn33 did not react to the detergent-insoluble fractions (Fig. 7c, d). These results show that tau expression does not impact the levels of soluble oligomers recognized by Syn33 in our TgA53T model. Similar results were seen in our analysis of cortical and hippocampal tissues [4].

We also examined whether the αS pathology in the TgA53T mouse model was associated with obvious increases in pathological tau. However, our analysis for AT8, PHF1, and MC1 pathological tau showed that, even at the end stage, accumulation of hyperphosphorylated tau was not detected both biochemically and histologically (Additional file 1: Fig. S12g–l). Increased activation of GSK3β has been proposed as a mediator of αS-induced neuronal dysfunction [5, 7, 26, 27]. Thus, we also examined spinal cord lysates for the levels of active GSK3β, as measured by phosphorylated Tyr-216 (pY216) [28]. Our results showed that neither total GSK3β levels nor pY216-GSK3β activation was increased as a function of αS pathology, or was impacted by tau expression (Additional file 1: Fig. S12a–f).

### Endoplasmic reticulum stress (ERS) and autophagy pathway protein clearance

We previously showed that α-synucleinopathy in the TgA53T model is associated with chronic ERS [14] and dysfunctions in the autophagy-lysosomal pathway (ALP) [15]. Because both ERS and ALP deficits follow the onset of αS pathology, we examined whether ERS and ALP in the TgA53T model are affected in a tau-dependent manner. We performed biochemical (Western blot) analysis in 70-dpi and end-stage spinal cord lysates for markers of ERS and ALP (Additional file 1: Fig. S13**)**. While there was no obvious indication of ALP abnormalities at 70 dpi (Additional file 1: Fig. S13a), analysis of spinal cord lysates from end-stage TgA53T mice showed expected ALP abnormalities (Additional file 1: Fig. S13b). ALP markers LC3 II/I ratio, p62, and pAMPK/AMPK ratio were not different between TgA53T and TgA53T/mTau^−/−^ mice.

Analysis of ERS markers Grp78 and p-eIF2α/eIF2α ratio showed expected signs of chronic ERS in end-stage TgA53T and TgA53T/mTau^−/−^ mice (Additional file 1: Fig. S13b). While the level of Grp78 was similarly increased in both TgA53T and TgA53T/mTau^−/−^ mice, TgA53T/mTau^−/−^ had modest but significantly reduced p-eIF2α/eIF2α ratio compared to TgA53T. No signs of ERS were seen in lysates from 70-dpi subjects (Additional file 1: Fig. S13a). Similar results for ALP and ERS markers were observed in brainstem lysates from 70-dpi and end-stage animals (Additional file 1: Fig. S14). There were no signs of change in ERS or ALP in the brainstem of 70-dpi animals (Additional file 1: Fig. S14a). However, in the brainstem, there were elevated ALP markers at the end stage as previously described [15], but no sign of ERS in the end-stage subjects (Additional file 1: Fig. S14b).

Collectively, our results show that both ERS and ALP deficits associated with α-synucleinopathy in the TgA53T mouse model occur after the αS pathology is well developed. Further, unlike the loss of motor neurons (Fig. 6), both ERS and ALP deficits seem to be independent of tau expression.

### Tau expression does not affect αS PFF uptake or processing in neurons but prevents PFF-induced neurotoxicity

Our in vivo studies using the TgA53T model show that the loss of tau expression leads to a delay in αS aggregation, inflammation, and neurodegeneration. However, it is unknown if the loss of tau expression affects early processes related to the development of αS pathology. Moreover, if loss of tau allows neurons to be more resistant to the toxic effects of αS pathology, neurons may be able to more efficiently clear abnormal αS. Thus, we determined whether tau modulates the initiation of αS pathology and αS-dependent neurodegeneration in a cell autonomous manner. To address this, we established primary neuronal cultures from wild-type (nTg) and mTau^−/−^ mice and exposed the neurons to αS PFF.

To determine whether tau mediates the initiation and progression of intraneuronal αS pathology following PFF exposure, we examined the neuronal uptake of WT αS PFF in primary hippocampal neurons cultured from nTg and mTau^−/−^ mice. Cultured neurons were exposed to αS PFF for 2 h. After this 2-h incubation, PFF-containing medium was removed, neurons were washed with PFF-free medium to remove any extracellular PFF, and then fresh PFF-free medium was added. Neuronal lysates were then collected at 0, 3, 6, 16, 24 and 48 h following the 2-h incubation with αS PFFs. As expected, neurons rapidly internalized αS PFF and the internalized αS accumulated as a truncated protein [29] (Additional file 1: Fig. S15a, b). Quantitative analysis of uptake, truncation, and clearance of αS PFF showed that the nTg and mTau^−/−^ neurons metabolized exogenous PFF almost identically (Additional file 1: Fig. S15a, b).

**Fig. 8:**
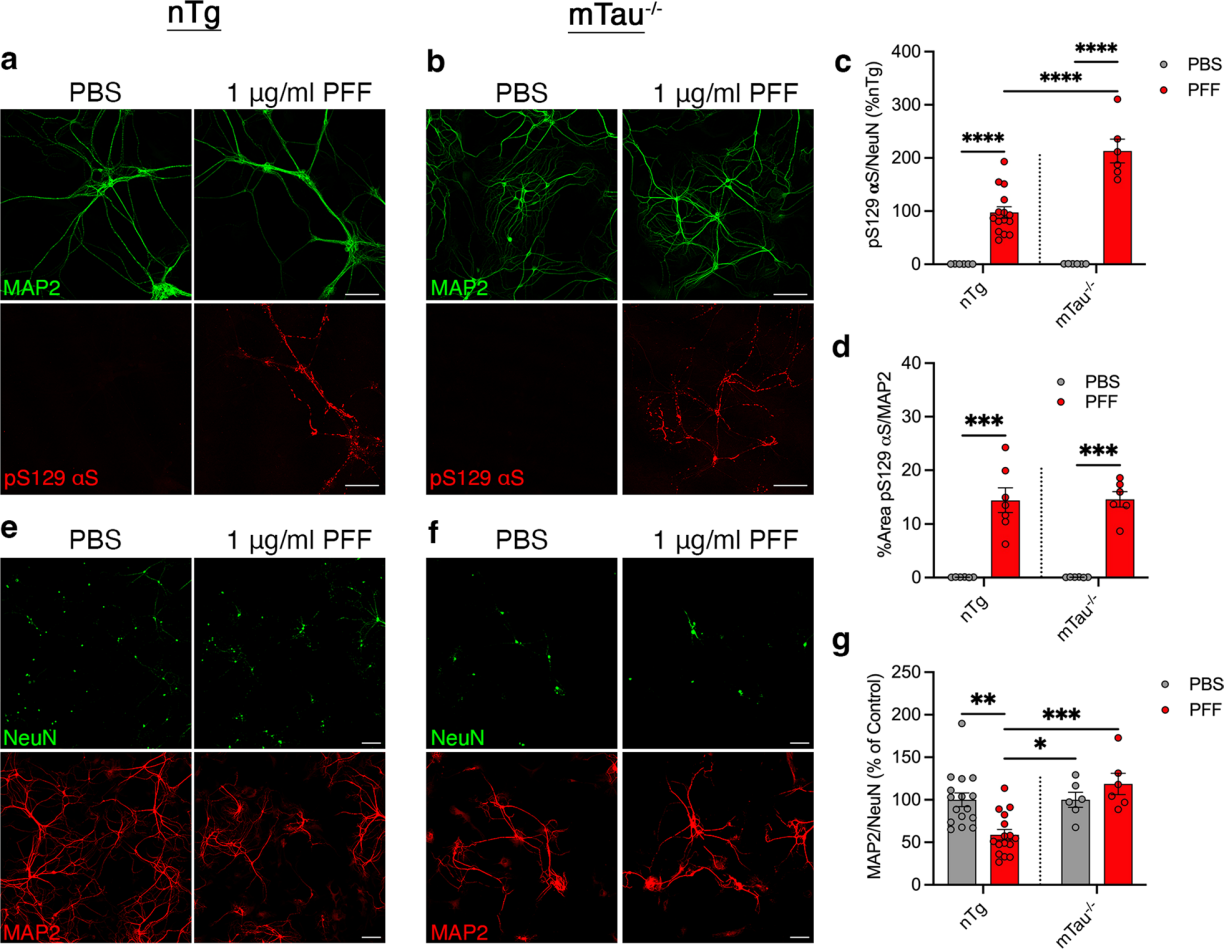
Neurons lacking tau expression are protected from αS PFF-induced loss of dendrites without impacting the development of pS129 αS+ aggregates. **a**, **b** Primary mouse hippocampal neurons from nTg (**a**) and mTau^−/−^ (**b**) were induced to develop αS pathology by αS PFF treatment. Same levels of pS129 αS+ aggregates, normalized to MAP2 area,  were seen in nTg (**c**; *P* = 0.0001, PBS vs PFF) and mTau^−/−^ (**c**; *P* = 0.0002, PBS vs PFF) neurons 14 days after PFF treatment. pS129 αS+ aggregates normalized to NeuN demonstrated a significant difference between nTg and mTau^−/−^ (**d**; *P* < 0.0001, PBS vs PFF for both nTg and mTau^−/−^, and nTg PFF vs mTau^−/−^ PFF). Analysis of MAP2+ neurites/dendrites at 14 days post-PBS or αS PFF addition to nTg (**e**) and mTau^−/−^ (**f**) neurons. αS PFF treatment led to significant loss of MAP2 area in nTg neurons (**e, g**; *P* = 0.0010, PBS vs PFF; *P* = 0.0004, nTg PFF vs mTau^−/−^ PFF; *P* = 0.0230, nTg PFF vs mTau^−/−^ PBS) but not mTau^−/−^ primary hippocampal neurons (**f, g**; *P* = 0.8297, PBS vs PFF), compared to PBS controls. Abbreviations: phosphate buffered saline, PBS; preformed fibril, PFF; Two-way ANOVA with Sidak’s multiple comparisons test; *P < 0.05, **P < 0.01, and ***P < 0.001. Scale bars = 100 μm;  *n* = 6–16 randomly selected areas from 3 to 5 independent cultures; error bars represent mean ± SEM

We next investigated whether tau expression affects αS aggregation and neuronal survival following αS PFF exposure. Primary nTg mouse hippocampal neurons were exposed to αS PFF and evaluated for the presence of pathological pS129 αS and neurodegeneration 14 days post PFF exposure in vitro. αS PFF applied to primary hippocampal neurons in vitro led to a dose-dependent increase in pS129 αS accumulation at 14 days post-PFF in the absence of significant NeuN^+^ neuronal loss (Additional file 1: Fig. S15c–e). To determine if tau expression modulates PFF-induced αS aggregation in neurons, we examined the levels of pS129 αS in nTg and mTau^−/−^ cultures exposed to PFF treatment. To account for possible differences in the density of neurons and/or neurites between the cultures, the area of pS129 αS staining was normalized to NeuN^+^ cells or the MAP2-stained area. When normalized to NeuN, Tau^−/−^ neurons exhibited higher pS129αS staining than the nTg neurons and a significant difference existed between PFF-treated nTg and mTau^−/−^ neurons (Fig. 8c). When normalized to MAP2-stained area, there was no difference in pS129 αS accumulation between nTg and mTau^−/−^ neurons at 14 days post-PFF (Fig. 8a, b, d). The addition of αS PFF did not induce loss of NeuN^+^ neurons in culture (Additional file 1: Fig. S15c, d) but did induce progressive loss of MAP2-stained dendrites (Fig. 8e, g; Additional file 1: Fig. S16). Specifically, αS PFF-induced αS aggregation in nTg neurons led to simplification of dendritic arborization, as indicated by reduced MAP2-stained area per NeuN^+^ cell at 14 days post-PFF (Fig. 8e, g; Additional file 1: Fig. S16). Significantly, in mTau^−/−^ neurons, the loss of MAP2 at 14 days post-PFF was prevented (Fig. 8f, g). These results show that the loss of mTau expression attenuates the PFF-induced neuronal toxicity without decreasing the neuronal accumulation of pathological αS.

## Discussion

Through in vitro and in vivo studies examining the effect of expression of endogenous mouse tau on αS pathology and α-synucleinopathy pathophysiology, we unequivocally demonstrate that tau has a central role in α-synucleinopathy disease progression. Previously, we showed that mutant αS causes synaptic and memory deficits in a tau-dependent manner [4, 5]. Here, we extend our previous studies by showing that the onset of motor dysfunction and reduced life-span of TgA53T mice were attenuated by the loss of endogenous tau expression. While the removal of tau significantly delayed the onset of disease and disease progression to end stage, we did not observe any differences in αS aggregation and neuropathology in mice at 40 dpi and end stage. However, analysis of TgA53T mice at an intermediate time point (70 dpi) revealed that while tau expression did not impact the amount of αS aggregates, loss of tau was associated with reduced neuropathology, including reduced motor neuron degeneration. Possibly, immunohistochemistry may not be sensitive enough to identify smaller changes in aggregates and levels of pS129 αS in white matter and in non-neuronal cells [25]. Thus, it is possible that the loss of tau expression leads to smaller pS129 αS structures that are not readily visible by light microscopy or selectively reduce accumulation of pS129 αS in larger neurons. This difference in αS-associated neuropathology as a function of tau expression is consistent with the fact that the lack of tau expression reverses several motor deficits associated with our TgA53T model. Considering the delay in disease onset in TgA53T/mTau^−/−^ mice, it is likely that the overall neuropathology was normalized upon reaching end stage between TgA53T and TgA53T/mTau^−/−^ mice. Thus, analysis of all groups at the same time point following initial αS PFF intramuscular inoculation revealed the temporal difference in the onset and progression of neuropathology and neurodegeneration.

While correlative studies with the TgA53T model establish that tau expression is a significant pathological contributor to α-synucleinopathy in vivo, the overall onset and progression of α-synucleinopathy likely involve complex interactions between different cell types as well as the homeostatic condition of the neuron. Thus, while we cannot conclusively determine if tau expression directly impacts αS pathology or if tau acts downstream of αS pathology in vivo, our studies using αS PFF-treated primary hippocampal neurons show that tau expression does not impact the PFF-mediated accumulation of pathological αS species including pS129 αS. However, the lack of tau expression was able to completely reverse the neurotoxic effects of PFF-induced pS129 αS in neurons in vitro. Thus, we conclude that tau is important for mediating the neurotoxic effects associated with αS pathology without directly affecting the generation of αS pathology.

Currently, there is some controversy regarding the role of tau in α-synucleinopathy. Our study suggests that while there may be a mild delay of αS pathological progression as a function of tau expression, a robust delay in neuronal toxicity may better explain the observed delay in disease onset and behavioral deficits observed in the TgA53T/mTau^−/−^ mice. Likewise, our conclusion that tau does not impact the onset and progression of αS pathology is supported by recent studies using αS PFF inoculation mouse models [6, 11]. In these studies, no differences in αS pathology were seen following hippocampal [6] or intrastriatal [11] injections of mouse αS PFF to nTg and mTau^−/−^ mice. However, these studies did not determine if the behavioral deficits or neurodegeneration was attenuated in mTau^−/−^ mice [6] or only examined loss of nigral neurons at end stage [11]. In our study, the lack of tau-dependent pathological differences at 40 dpi as well as at end stage, but significant delay in neurodegeneration and pathological markers at 70 dpi, suggests that tau may act downstream of overt αS pathology, affecting neuronal survival. In contrast, another recent study presented evidence that removal of toxic tau oligomers in vivo could reduce αS pathology and insoluble αS oligomers in the M83 TgA53T model, indicating reciprocal pathological interactions between αS pathology and tau [10]. Specifically, the hypothesis is that αS pathology induces tau oligomers, which in turn promote αS pathology and neurological abnormalities. However, our analysis showed that the absence of tau expression did not reduce the abundance of soluble αS oligomers detected by Syn33 (off-pathway) or SDS-stable high-molecular-weight αS oligomers. Based on our results, we favor the notion that the endogenous tau expression does not directly impact αS oligomer levels or pathology. We note that the discrepancy between our study and that by Gerson et al. [10] is in the involvement of tau oligomers in αS pathology. However, both studies agree that reduction in tau will attenuate αS toxicity. We recognize that multiple factors could contribute to the differences in the results, including background strain of G2-3 vs M83 mice, and experimental paradigm (e.g. PFF-induced pathology compared to natural ageing paradigm used by Gerson et al. [10]). While mouse tau may also have less of an interaction with human αS, previous studies using mouse αS fibrils in WT or mTau^−/−^ mice found that αS pathology was independent of tau expression [6, 11]. In addition, unlike studies that have observed αS aggregates to cross-seed tau pathology in tau-overexpressing mice and visa versa [30], we did not observe any biochemical or histological tau abnormalities as a result of TgA53T overexpression in mice with endogenous levels of tau. Similarly, in vitro application demonstrating polymerization rates of A53T vs WT human αS and the ability to co-polymerize tau [31] may also require specific expression ratios of each protein when applied in vivo. Another possibility for the lack of tau pathology may be a lower efficiency of human αS to cause endogenous mouse tau fibrillization.

Our lab has previously demonstrated that HuαS^A53T^ overexpression leads to tau-dependent post-synaptic deficits through the GSK3β-dependent phosphorylation of tau, leading to mislocalization of tau and increased AMPA receptor internalization [5]. Here, we demonstrated that exogenous αS PFF leads to dendritic simplification in nTg neurons, as well as motor neuron degeneration in TgA53T mice, which are protected and delayed, respectively, with tau loss. However, given that removing tau expression does not impact the levels of total or active GSK3β, the tau-dependent neuroprotection seen here does not require alterations in the state of GSK3β activation.

## Conclusions

The current study represents a significant and important development in the relationship between αS and tau-dependent disease progression. We provide evidence that the PFF-induced disease progression can be delayed with the removal of tau. In addition, while tau removal led to modest reductions in αS-mediated pathological changes in vivo, it did lead to significant improvements in spinal motor neuron survival as well as morphological markers in vitro.

Collectively, our data suggest that tau may act downstream of αS pathology to affect neuronal homeostasis and survival in α-synucleinopathies. These results support the use of therapeutic strategies that aim to reduce the overall tau expression levels for treatment of α-synucleinopathies, including the current Phase I clinical trial by Biogen/Ionis utilizing antisense oligonucleotides to directly impact tau expression levels. Taken together, this work provides important insight into the tau-dependent mechanisms in neurodegenerative diseases, critical to the advancement of much needed disease-modifying therapies.

## Supplementary Information

**Additional file 1: Table S1.** List of antibodies utilized in experiments. **Fig. S1.** Open field analysis. **Fig. S2.** Levels of αS in Triton-X100 soluble and insoluble spinal cord lysates from end-stage mice. **Fig. S3.** Biochemical analysis of brainstem in end stage and 70 dpi mice. **Fig. S4.** Representative low magnification images of end stage spinal cord histology (see analysis in Fig. 3). **Fig. S5.** Histological analysis of spinal cord from αS PFF injected, and age-matched, nTg and TgA53T mice. **Fig. S6.** Qualitative images of brainstem, cerebellum, CA1, and cortex in end-stage mice. **Fig. S7.** Triton-X100 soluble and insoluble spinal cord lysates from 70 dpi mice. **Fig. S8.** Representative low magnification images of 70 dpi spinal cord histology (see analysis in Fig. 5). **Fig. S9.** Qualitative images of brainstem, cerebellum, CA1, and cortex in 70 dpi mice. **Fig. S10.** Biochemical and histological analysis in 40 dpi tissues. **Fig. S11.** Representative low magnification images of 70 dpi and end stage spinal cord NeuN histology (see analysis in Fig. 6). **Fig. S12.** Analysis of GSK3β expression in 70 dpi and end stage spinal cords. **Fig. S13.** Endoplasmic reticulum stress and autophagy pathway protein clearance pathways deficits in TgA53T mice are not affected by tau expression. **Fig. S14.** Endoplasmic reticulum stress and autophagy pathway protein clearance pathway analysis in 70 dpi and end stage brainstems. **Fig. S15.** PFF uptake and processing in primary neurons is not affected by tau expression. **Fig. S16.** PFF leads to simplification of dendritic morphology after 14 dpi in nTg neurons in vitro.

## Data Availability

Any additional data and materials are available from corresponding author on reasonable request.

## Abbreviations

αS: α-Synuclein
HuαS: Human αS
TgA53T: Transgenic A53T mutant synuclein
PFF: Preformed fibril
nTg: Non-transgenic
dpi: Days post inoculation
PD: Parkinson’s disease
SNpc: Substantia Nigra pars compacta
LB: Lewy body
LN: Lewy neurite
mTau^−^^/^^−^: Mouse tau knockout
PBS: Phosphate buffered saline
PFA: Paraformaldehyde
TX-100: Triton-100
GSK3β: Glycogen synthase kinase 3β
pY216: Phosphorylated tyrosine 216
ERS: Endoplasmic reticulum stress
ALP: Autophagy lysosome pathway

## References

[1] Braak H, Tredici KD, Rüb U, de Vos RAI, Jansen Steur ENH, Braak E (2003). Staging of brain pathology related to sporadic Parkinson’s disease. Neurobiol Aging.

[2] Del Tredici K, Rüb U, de Vos RAI, Bohl JRE, Braak H (2002). Where does Parkinson disease pathology begin in the brain?. J Neuropathol Exp Neurol.

[3] Lee MK, Stirling W, Xu Y, Xu X, Qui D, Mandir AS (2002). Human α-synuclein-harboring familial Parkinson’s disease-linked Ala-53 → Thr mutation causes neurodegenerative disease with α-synuclein aggregation in transgenic mice. Proc Natl Acad Sci USA.

[4] Singh B, Covelo A, Martell-Martínez H, Nanclares C, Sherman MA, Okematti E (2019). Tau is required for progressive synaptic and memory deficits in a transgenic mouse model of α-synucleinopathy. Acta Neuropathol.

[5] Teravskis PJ, Covelo A, Miller EC, Singh B, Martell-Martínez HA, Benneyworth MA (2018). A53T mutant alpha-synuclein induces tau-dependent postsynaptic impairment independently of neurodegenerative changes. J Neurosci.

[6] Bassil F, Meymand ES, Brown HJ, Xu H, Cox TO, Pattabhiraman S (2021). α-Synuclein modulates tau spreading in mouse brains. J Exp Med.

[7] Haggerty T, Credle J, Rodriguez O, Wills J, Oaks AW, Masliah E (2011). Hyperphosphorylated Tau in an α-synuclein-overexpressing transgenic model of Parkinson’s disease: Tauopathy in transgenic mice and PD. Eur J Neurosci.

[8] Khandelwal PJ, Dumanis SB, Herman AM, Rebeck GW, Moussa CE-H (2012). Wild type and P301L mutant Tau promote neuro-inflammation and α-Synuclein accumulation in lentiviral gene delivery models. Mol Cell Neurosci.

[9] Morris M, Koyama A, Masliah E, Mucke L (2011). Tau reduction does not prevent motor deficits in two mouse models of Parkinson’s disease. PLoS ONE.

[10] Gerson JE, Farmer KM, Henson N, Castillo-Carranza DL, Carretero Murillo M, Sengupta U (2018). Tau oligomers mediate α-synuclein toxicity and can be targeted by immunotherapy. Mol Neurodegener.

[11] Stoyka LE, Mahoney CL, Thrasher DR, Russell DL, Cook AK, Harris AT, et al. Templated α-synuclein inclusion formation is independent of endogenous tau. eNeuro. 2021;8:ENEURO.0458-20.2021. 10.1523/ENEURO.0458-20.2021 PMC8213444 33972291

[12] Sacino AN, Brooks M, Thomas MA, McKinney AB, Lee S, Regenhardt RW (2014). Intramuscular injection of -synuclein induces CNS α-synuclein pathology and a rapid-onset motor phenotype in transgenic mice. Proc Natl Acad Sci USA.

[13] Tucker KL, Meyer M, Barde YA (2001). Neurotrophins are required for nerve growth during development. Nat Neurosci.

[14] Colla E, Jensen PH, Pletnikova O, Troncoso JC, Glabe C, Lee MK (2012). Accumulation of toxic -synuclein oligomer within endoplasmic reticulum occurs in -synucleinopathy in vivo. J Neurosci.

[15] Karim MdR, Liao EE, Kim J, Meints J, Martinez HM, Pletnikova O (2020). α-Synucleinopathy associated c-Abl activation causes p53-dependent autophagy impairment. Mol Neurodegener.

[16] Martin LJ, Pan Y, Price AC, Sterling W, Copeland NG, Jenkins NA (2006). Parkinson’s disease α-synuclein transgenic mice develop neuronal mitochondrial degeneration and cell death. J Neurosci.

[17] Liu Y, Yoo M-J, Savonenko A, Stirling W, Price DL, Borchelt DR (2008). Amyloid pathology is associated with progressive monoaminergic neurodegeneration in a transgenic mouse model of Alzheimer’s disease. J Neurosci.

[18] Volpicelli-Daley LA, Luk KC, Lee VM-Y (2014). Addition of exogenous α-synuclein preformed fibrils to primary neuronal cultures to seed recruitment of endogenous α-synuclein to Lewy body and Lewy neurite–like aggregates. Nat Protoc.

[19] Matsuura K, Kabuto H, Makino H, Ogawa N (1997). Pole test is a useful method for evaluating the mouse movement disorder caused by striatal dopamine depletion. J Neurosci Methods.

[20] Colla E, Coune P, Liu Y, Pletnikova O, Troncoso JC, Iwatsubo T (2012). Endoplasmic reticulum stress is important for the manifestations of -synucleinopathy in vivo. J Neurosci.

[21] Li W (2004). Stabilization of -synuclein protein with aging and familial Parkinson’s disease-linked A53T mutation. J Neurosci.

[22] Li W, West N, Colla E, Pletnikova O, Troncoso JC, Marsh L (2005). Aggregation promoting C-terminal truncation of -synuclein is a normal cellular process and is enhanced by the familial Parkinson’s disease-linked mutations. Proc Natl Acad Sci USA.

[23] Li W, Lee MK (2005). Antiapoptotic property of human α-synuclein in neuronal cell lines is associated with the inhibition of caspase-3 but not caspase-9 activity: Antiapoptotic property of human α-synuclein. J Neurochem.

[24] Khan SS, LaCroix M, Boyle G, Sherman MA, Brown JL, Amar F (2018). Bidirectional modulation of Alzheimer phenotype by alpha-synuclein in mice and primary neurons. Acta Neuropathol.

[25] Sorrentino ZA, Giasson BI, Chakrabarty P (2019). α-Synuclein and astrocytes: tracing the pathways from homeostasis to neurodegeneration in Lewy body disease. Acta Neuropathol.

[26] Kawakami F, Suzuki M, Shimada N, Kagiya G, Ohta E, Tamura K (2011). Stimulatory effect of α-synuclein on the tau-phosphorylation by GSK-3β: α-Synuclein induced hyperphosphorylation of tau. FEBS J.

[27] Wills J, Credle J, Haggerty T, Lee JH, Oaks AW, Sidhu A (2011). Tauopathic changes in the striatum of A53T α-synuclein mutant mouse model of Parkinson’s disease. PLoS ONE.

[28] Krishnankutty A, Kimura T, Saito T, Aoyagi K, Asada A, Takahashi SI (2017). In vivo regulation of glycogen synthase kinase 3β activity in neurons and brains. Sci Rep.

[29] Mahul-Mellier AL, Burtscher J, Maharjan N, Weerens L, Croisier M, Kuttler F (2020). The process of Lewy body formation, rather than simply α-synuclein fibrillization, is one of the major drivers of neurodegeneration. Proc Natl Acad Sci USA.

[30] Williams T, Sorrentino Z, Weinrich M, Giasson BI, Chakrabarty P (2020). Differential cross-seeding properties of tau and α-synuclein in mouse models of tauopathy and synucleinopathy. Brain Commun.

[31] Kotzbauer P (2004). Fibrillization of α-synuclein and tau in familial Parkinson’s disease caused by the A53T ?-synuclein mutation. Exp Neurol.

