# Supplementary material for: Loss of tau expression attenuates neurodegeneration associated with α-synucleinopathy
Source: Transl Neurodegener. 2022 Jul 1;11:34. doi: 10.1186/s40035-022-00309-x (PMC9248195; doi:10.1186/s40035-022-00309-x)
Supplement: Supplementary file 1 — Additional file 1: Table S1. List of antibodies utilized in experiments. Fig. S1. Open field analysis. Fig. S2. Levels of αS in Triton-X100 soluble and insoluble spinal cord lysates from end-stage mice. Fig. S3. Biochemical analysis of brainstem in end stage and 70 dpi mice. Fig. S4. Representative low magnification images of end stage spinal cord histology (see analysis in Fig. 3). Fig. S5. Histological analysis of spinal cord from αS PFF injected, and age-matched, nTg and TgA53T mice. Fig. S6. Qualitative images of brainstem, cerebellum, CA1, and cortex in end-stage mice. Fig. S7. Triton-X100 soluble and insoluble spinal cord lysates from 70 dpi mice. Fig. S8. Representative low magnification images of 70 dpi spinal cord histology (see analysis in Fig. 5). Fig. S9. Qualitative images of brainstem, cerebellum, CA1, and cortex in 70 dpi mice. Fig. S10. Biochemical and histological analysis in 40 dpi tissues. Fig. S11. Representative low magnification images of 70 dpi and end stage spinal cord NeuN histology (see analysis in Fig. 6). Fig. S12. Analysis of GSK3β expression in 70 dpi and end stage spinal cords. Fig. S13. Endoplasmic reticulum stress and autophagy pathway protein clearance pathways deficits in TgA53T mice are not affected by tau expression. Fig. S14. Endoplasmic reticulum stress and autophagy pathway protein clearance pathway analysis in 70 dpi and end stage brainstems. Fig. S15. PFF uptake and processing in primary neurons is not affected by tau expression. Fig. S16. PFF leads to simplification of dendritic morphology after 14 dpi in nTg neurons in vitro. [file 40035_2022_309_MOESM1_ESM.docx]

**Additional File 1:**

**Supplementary Material**

Supplementary Table S1

Supplementary Figures S1-16

**Loss of tau expression attenuates neurodegeneration associated with α-synucleinopathy**

Scott C. Vermilyea^1,3^, Anne Christensen^1^, Joyce Meints^1,3^, Balvindar Singh^1^, Héctor Martell-Martínez^1,3^, Md. Razaul Karim^1,3^, Michael K. Lee^1,2,3*^

**Affiliations**

^1^Department of Neuroscience, University of Minnesota – Twin Cities, Minneapolis, MN, USA

^2^Institute for Translational Neuroscience; University of Minnesota – Twin Cities, Minneapolis, MN, USA

^3^Aligning Science Across Parkinson’s (ASAP) Collaborative Research Network, Chevy Chase, MD

| **Loading Controls** | **Company** | **Reference** | **Use** |
| --- | --- | --- | --- |
| GAPDH (D16H11) | Cell Signaling | 5174 | WB |
| 𝛂-tubulin | Abcam | 4074 | WB |
| Actin | MilliporeSigma | A2066 | DB |
| **𝛂-Synuclein Species** | **Company** | **Reference** | **Use** |
| 𝛂-Synuclein (total) | BD Transduction | 610787 | WB |
| Phospho Serine 129 𝛂S (pS129 𝛂S) | Abcam | Ab51253 | IHC,WB,ICC |
| Syn33 (conformation-specific oligomers) | Dr. Rakez Kayed (gift) | [1] | DB |
| 4D6 (total) | BioLegend | 834302 | DB |
| LB509 | Abcam | 27766 | DB |
| **Tau Species** | **Company** | **Reference** | **Use** |
| Tau5 (total) | Millipore | MAB361 | WB |
| AT8 | Invitrogen | MN1020 | WB |
| **Glial and Neuronal Markers** | **Company** | **Reference** | **Use** |
| Iba1 | Wako Chemical | 019-19741 | IHC |
| GFAP | Dako Cytomation | Z0334 | IHC |
| NeuN | Millipore | MAB377 | IHC,ICC |
| MAP2 | Abcam | Ab183830 | ICC |
| MAP2 | Millipore | MAB3418 | ICC |
| **Autophagy and ER Stress Pathways** | **Company** | **Reference** | **Use** |
| LC3 | Cell Signaling | 2775 | WB |
| p62 | Cell Signaling | 5114 | WB |
| pAMPK | Cell Signaling | 2535 | WB |
| AMPK (total) | Cell Signaling | 2532 | WB |
| Grp78 | Novus | NB300-520 | WB |
| p-eIF2𝛂 | Cell Signaling | 3398 | WB |
| eIF2𝛂 (total) | Cell Signaling | 5324 | WB |
| **GSK3𝛃 Species** | **Company** | **Reference** | **Use** |
| GSK3𝛃 (total) | Cell Signaling | 12456S | WB |
| Phospho Tyrosine GSK3𝛃 (pYGSK3𝛃) | Abcam | Ab75745 | WB |

**Table S1.** List of antibodies utilized in experiments. WB, western blot; DB, dot blot; IHC, immunohistochemistry; ICC, immunocytochemistry; ER, endoplasmic reticulum

1. Sengupta U, Guerrero-Muñoz MJ, Castillo-Carranza DL, Lasagna-Reeves CA, Gerson JE, Paulucci-Holthauzen AA, Krishnamurthy S, Farhed M, Jackson GR, Kayed R (2015) Pathological Interface Between Oligomeric Alpha-Synuclein and Tau in Synucleinopathies. Biological Psychiatry 78:672–683. doi: 10.1016/j.biopsych.2014.12.019


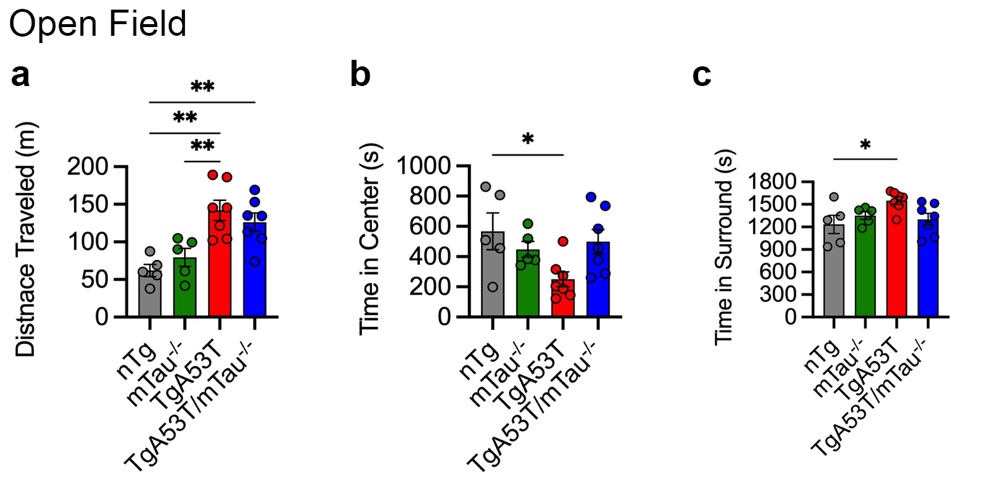


**Figure S1.** Open field analysis. **a** TgA53T mice, regardless of the tau genotype, are hyperactive compared to controls (F_(3,20)_ = 9.299, *p* = 0.0005). **b, c** Loss of tau reverses increased thigmotaxic behavior, a measure of increased anxiety, in TgA53T mice (F_(3,20)_ = 3.295, *p* = 0.0416). N = 5-7 animals/genotype. Error bars represent mean ± SEM

**
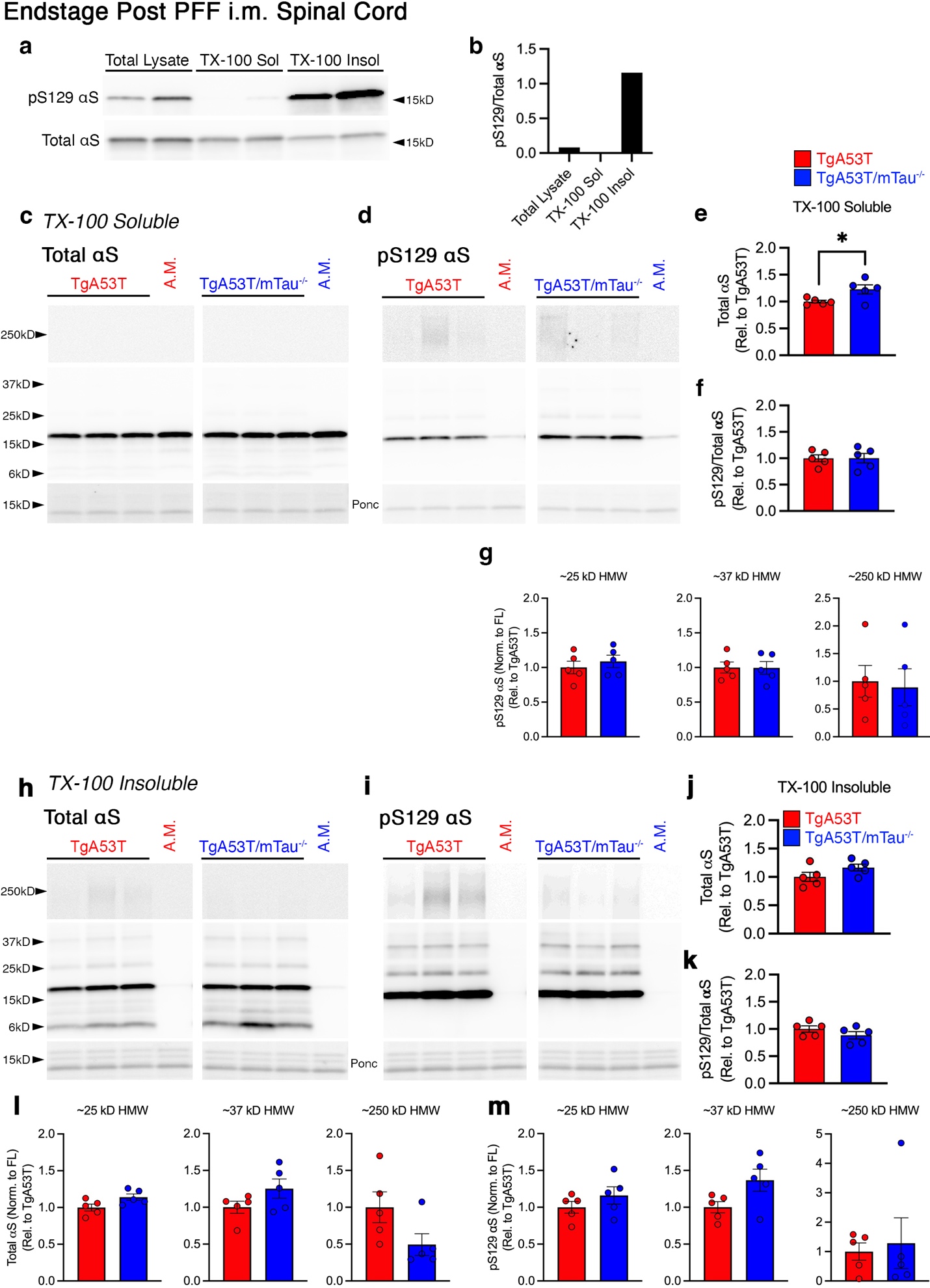
Figure S2.** Levels of αS in Triton-X100 soluble and insoluble spinal cord lysates from end stage mice. (**a, b**) Representative end stage spinal cord samples processed as total lysate, TX-100 soluble, and TX-100 insoluble fractions demonstrate the ratio of pS129 αS normalized to total αS is an order of magnitude less, and an order of magnitude higher in TX-100 soluble and insoluble fractions compared to total lysates respectively. All subsequent blots were exposed at sufficient levels for quantification and comparison of TgA53T and TgA53T/mTau^-/-^ samples. Immunoblot analysis for pS129 αS and total αS in Triton-X100 (TX-100) soluble and insoluble fractions from spinal cord show the abundance of αS are not affected by tau expression in end stage TgA53T mice. While TgA53T/mTau^-/-^ mice had higher total αS in TX-100 soluble fractions (**c, e**; *p* = 0.0358) compared to TgA53T mice, no difference was observed in pS129 αS (**d, f**), or in pS129 αS-positive SDS-stable αS oligomers (~25, 37, and ~250 kDa) (**g**; not observed when detecting total αS). Similarly, no difference in total or pS129 αS was observed in TX-100 insoluble fractions (**h-m**). All quantified bands were normalized to the respective ponceau S total protein. N = 5 animals/genotype. Abbreviations: age-matched control, A.M; High molecular weight, HMW; Ponceau S, Ponc. Analyses: *t*-test; **p* < 0.05; error bars represent mean ± SEM


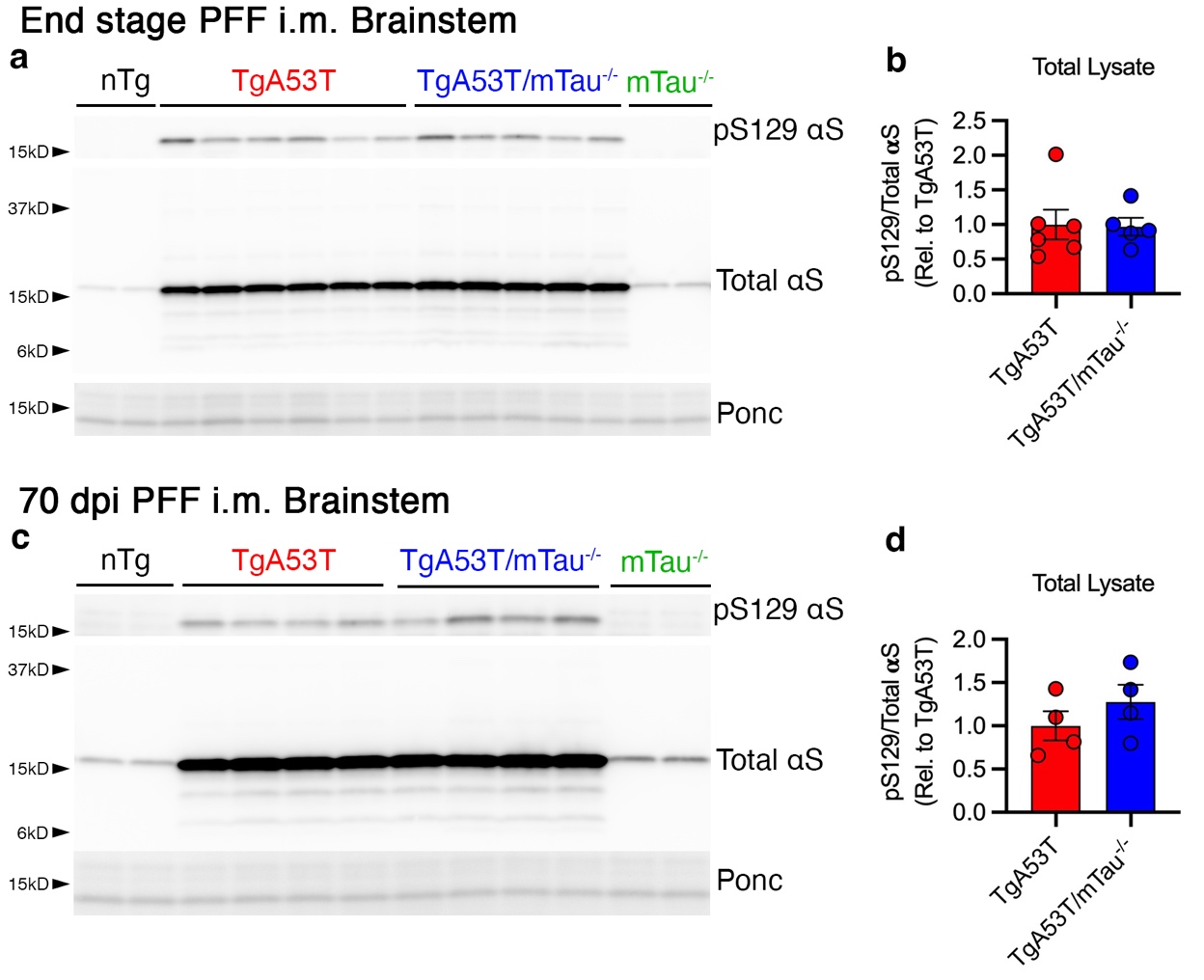


**Figure S3.** Biochemical analysis of brainstem in end stage and 70 dpi mice. Western blot of end stage (**a**) and 70 dpi (**c**) brainstem lysates show no difference in pS129 αS/total αS based on tau expression in TgA53T mice (**b, d**). All quantified bands were normalized to the respective ponceau S total protein. N = 4-6 animals/genotype. Abbreviations: days post inoculation, dpi; intramuscular, i.m.; arbitrary units, A.U.; Ponceau S, Ponc. Analyses: *t*-test. Error bars represent mean ± SEM


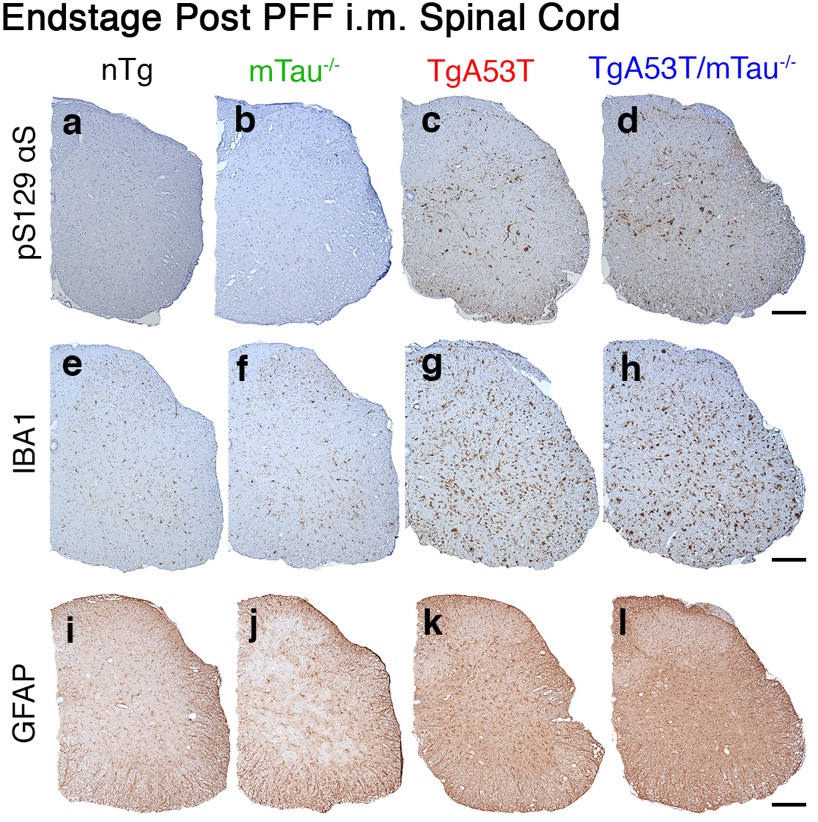


**Figure S4.** Representative low magnification images of end stage spinal cord histology (see analysis in Fig. 3). Representative hemi-spinal cord images of pS129 αS (**a-d**), Iba1 (**e-h**), and GFAP (**i-l**). Scale bars = 250 μm

**
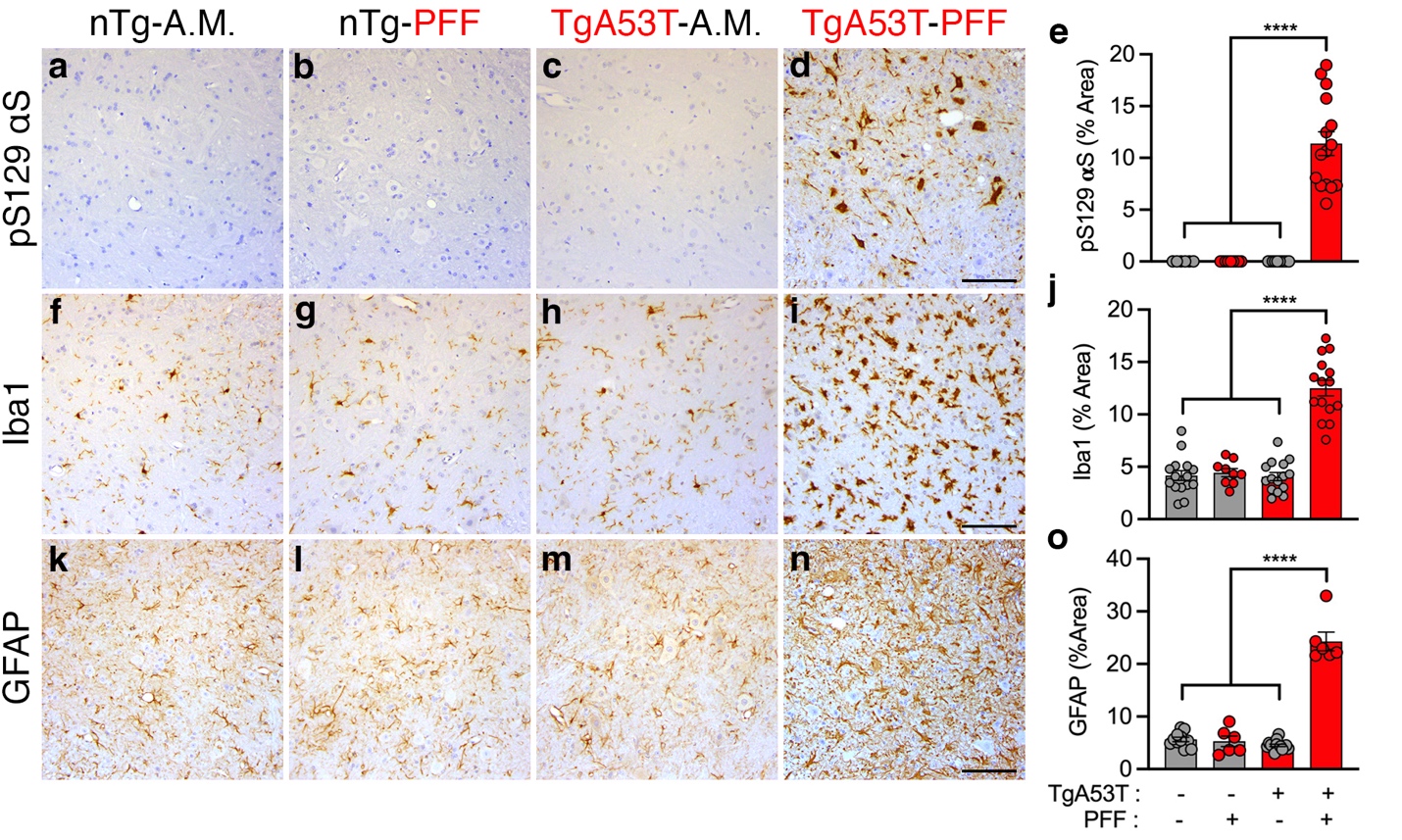
Figure S5.** Histological analysis of spinal cord from αS PFF injected, and age-matched, nTg and TgA53T mice. pS129 αS histopathology is only observed in TgA53T mice injected with PFF (**a-e**). nTg mice (age-matched and PFF injected) as well as age-matched TgA53T mice have equal abundance of Iba1 (**f-h, j**) and GFAP (**k-m, o**) in the grey matter of lumbar spinal cord. Only TgA53T mice injected with PFF have significantly higher neuroinflammation (i.e. increased Iba1 and GFAP; **i, j** and **n, o**) compared to all other conditions. nTg-PFF and TgA53T-PFF quantification also represented in Fig. 3. N = 6-15 sections from 3-5 animals/genotype. Abbreviations: age-matched, A.M; preformed fibril, PFF. Analyses: One-way ANOVA with Tukey’s posthoc analysis; ****p<0.0001; Scale bars = 100 μm; error bars represent mean ± SEM.


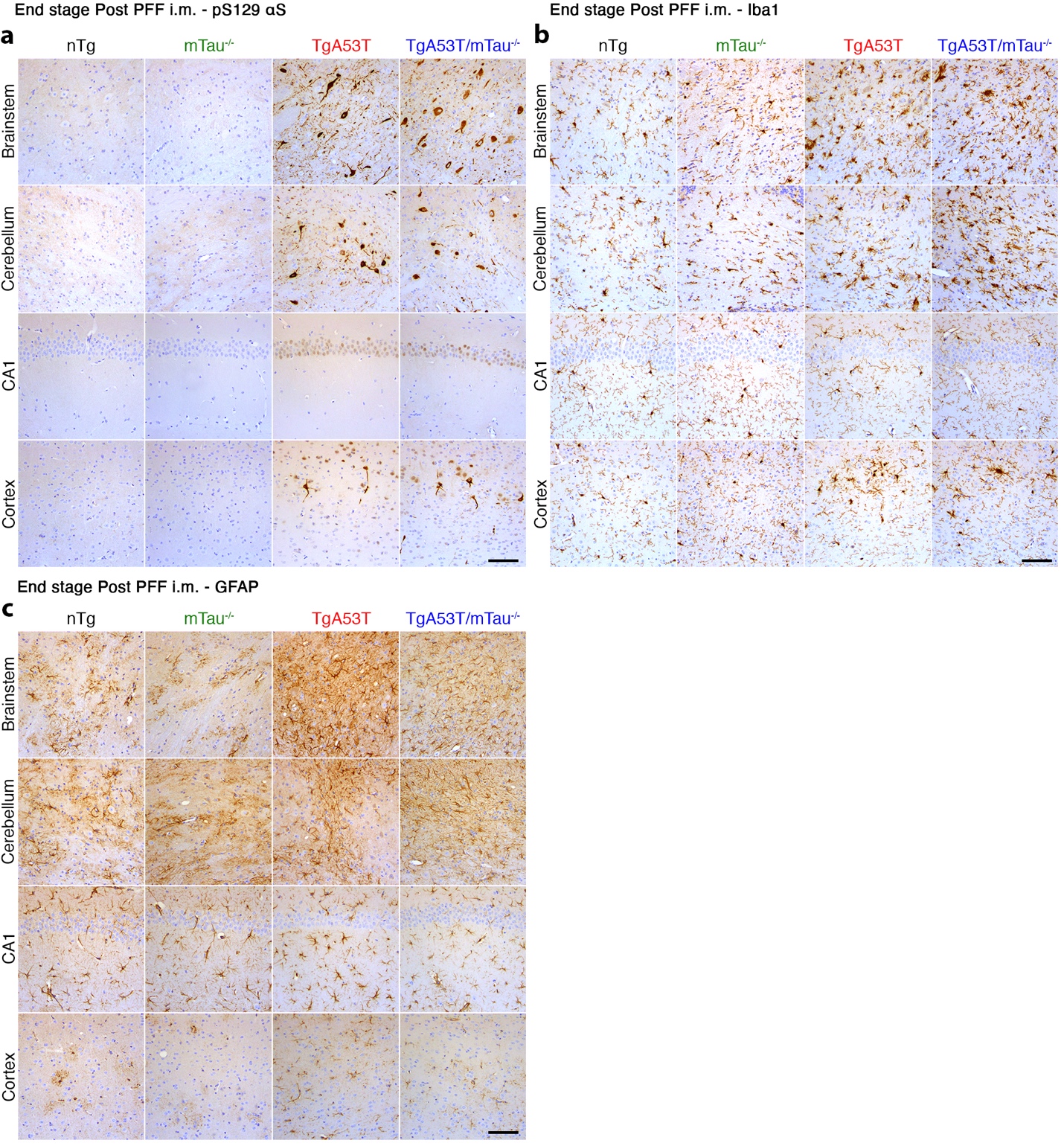


**Figure S6.** Qualitative images of brainstem, cerebellum, CA1, and cortex in end stage mice. **a** Representative pS129 αS pathology seen in brainstem, cerebellum, and cortex of TgA53T and TgA53T/mTau^-/-^. No qualitative differences were seen between of TgA53T and TgA53T/mTau^-/-^ mice. Inflammatory responses marked by Iba1 (**b**) and GFAP (**c**) showed similar results. Scale bars = 100 μm

**
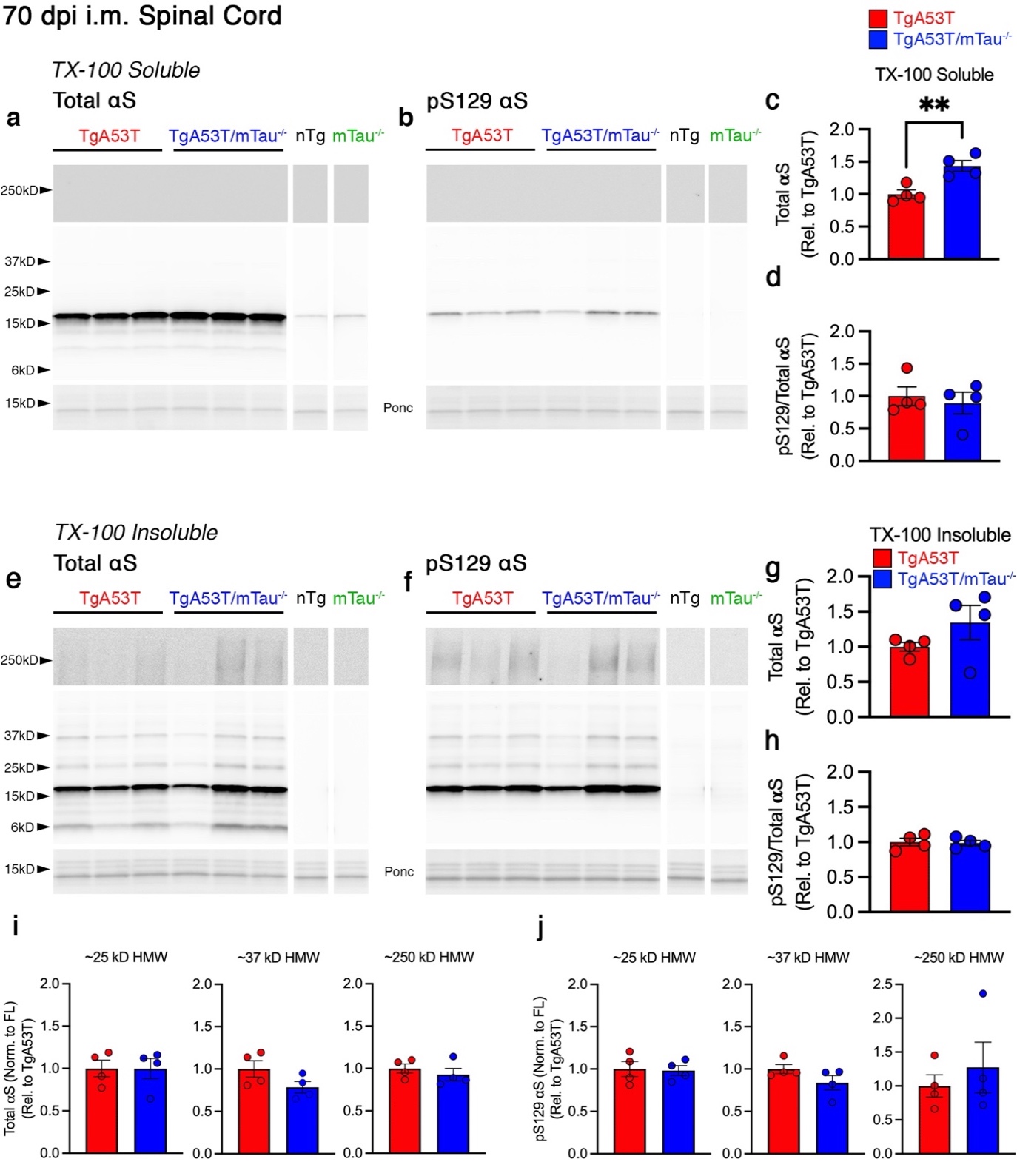
**

**Figure S7.** Triton-X100 soluble and insoluble spinal cord lysates from 70 dpi mice. Immunoblot analysis of Triton-X100 soluble and insoluble fraction spinal cords for total and pS129 αS. The abundance of pS129 αS in the fractions are not affected by tau expression in 70 dpi TgA53T mice. TgA53T/mTau^-/-^ mice had significantly higher total αS in TX-100 soluble fractions (**a, c**; *p* = 0.0059) compared to TgA53T mice. No difference was observed in pS129 αS (**b, d**), or in TX-100 insoluble fractions (**e-j**). All quantified bands were normalized to the respective ponceau S total protein. N = 4 animals/genotype. Abbreviations: High molecular weight, HMW; Ponceau S, Ponc. Analyses: *t*-test; ***p* < 0.01; error bars represent mean ± SEM.


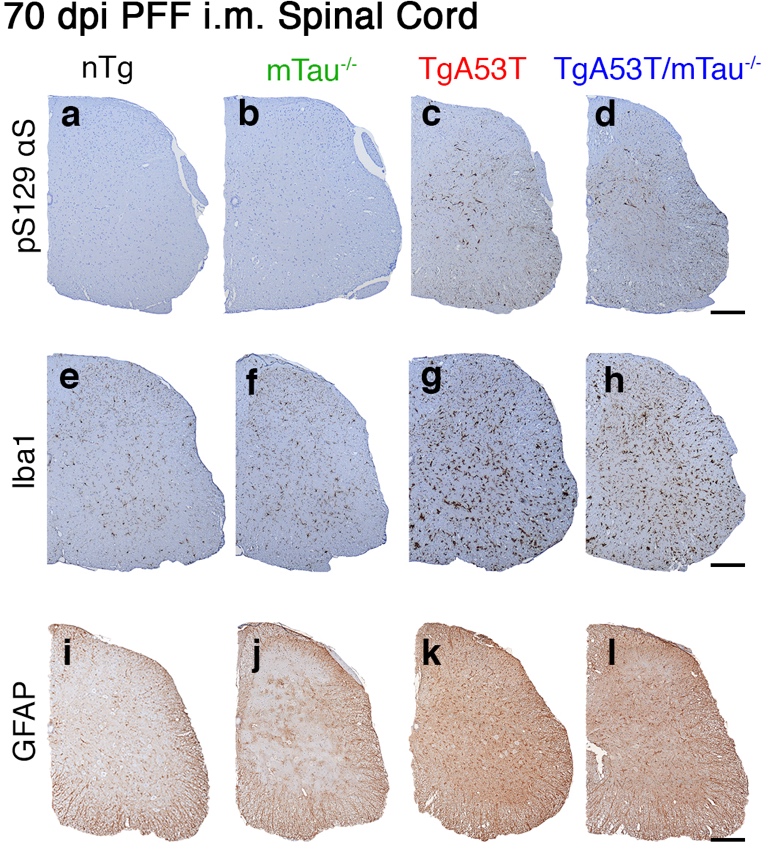


**Figure S8.** Representative low magnification images of 70 dpi spinal cord histology (see analysis in Fig. 5). Representative hemi-spinal cord images of pS129 αS (**a-d**), Iba1 (**e-h**), and GFAP (**i-l**). Scale bars = 250 μm

**
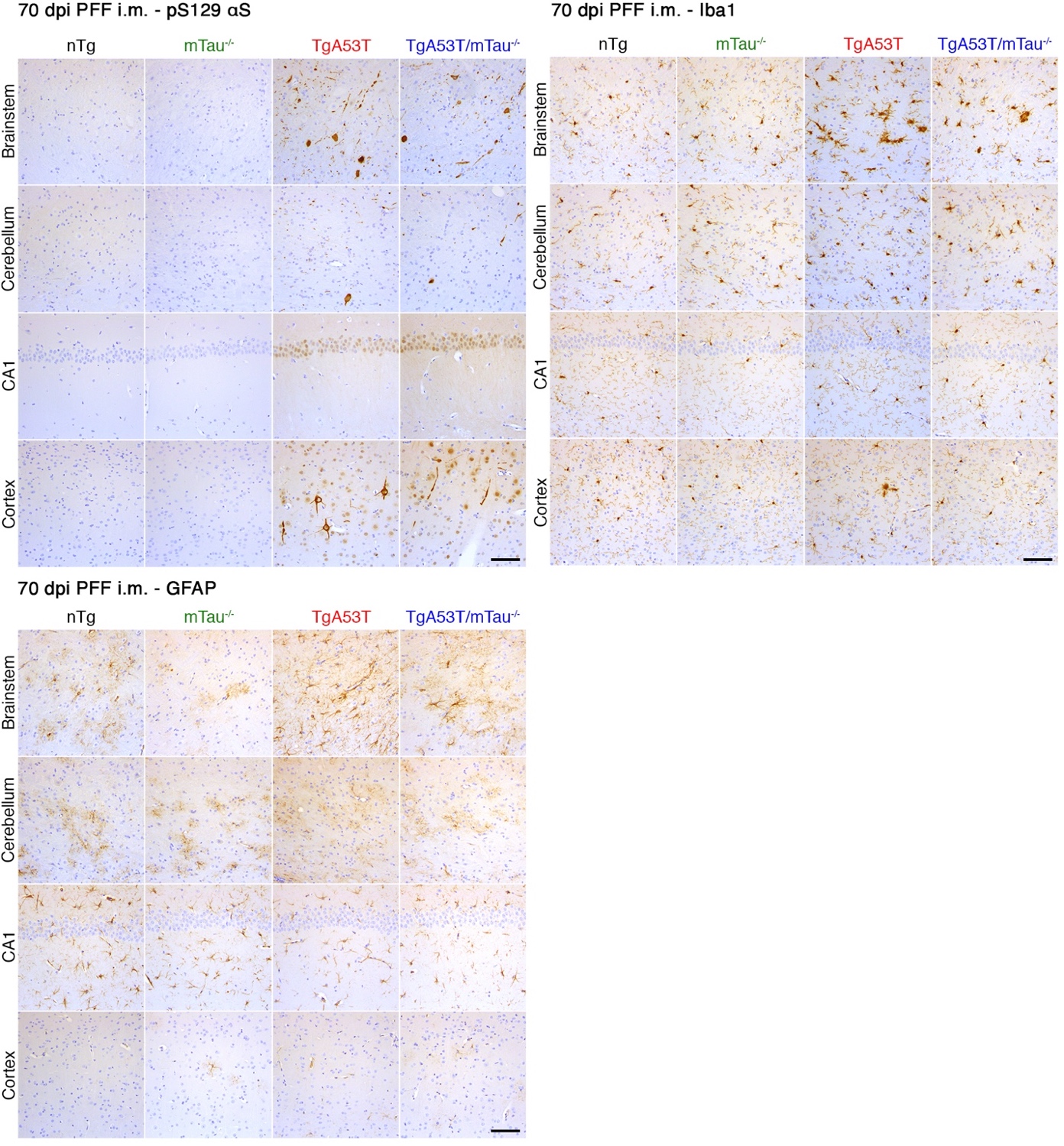
**

**Figure S9.** Qualitative images of brainstem, cerebellum, CA1, and cortex in 70 dpi mice. **a** pS129 αS pathology is present in brainstem, cerebellum, and cortex of TgA53T mice and was qualitatively similar to the quantitative results seen with spinal cord sections. No pS129 αS pathology was observed in the hippocampus. Inflammatory responses marked by Iba1 (**b**) and GFAP (**c**) showed similar results with most activation observed in brainstem regions. Scale bars = 100 μm

**
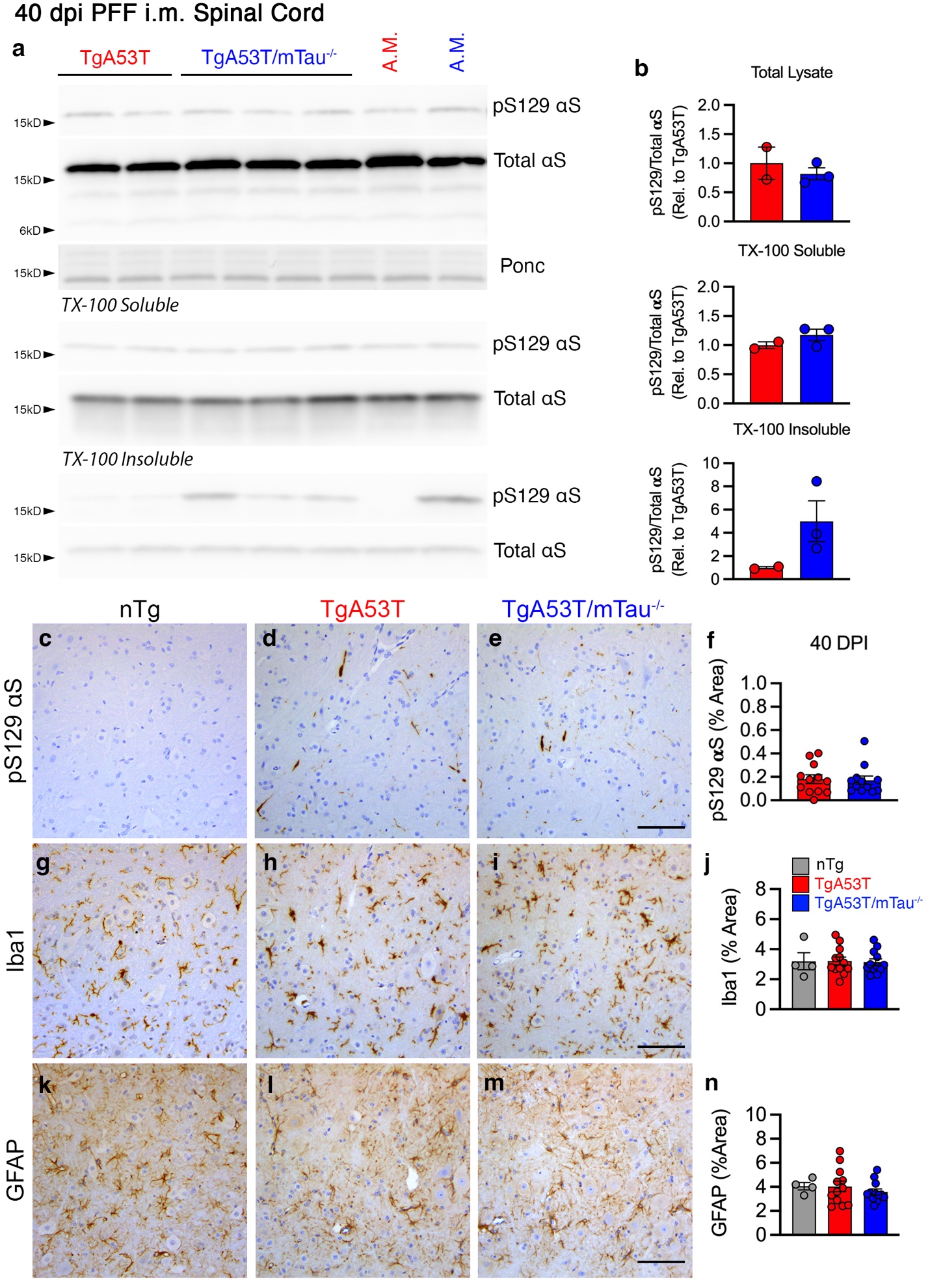
**

**Figure S10.** Biochemical and histological analysis in 40 dpi tissues. **a** & **b** Immunoblot analysis shows basal levels of pS129 αS in spinal cords from 40 dpi mice. Compared to age matched saline injected transgenic mice (A.M), there is no obvious increase in αS or pS129αS levels with PFF injection (TgA53T and TgA53T/mTau^-/-^). The blots showing TX100-insoluble pS129αS were highly overexpressed to show the pS129αS band and not quantitative. Graphs are shown for informative purpose and do not represent statistical analysis. All “quantified” bands were normalized to the respective ponceau S total protein. Histological analysis of pS129 αS (**c-f**) show that sparse pS129 αS aggregates can be seen at 40 dpi in TgA53T mice and that the amount of pS129 αS immunoreactivity was not different with or without tau expression (**f**). Similarly, no inflammatory activation was observed in microglia (**g-i**), and astrocytes (**k-m**), nor was there a difference between groups at 40 dpi (**j**, **n**). All quantified bands were normalized to the respective ponceau S total protein. N = 2-3 animals/genotype (immunoblot), N = 4-12 sections from 3-4 animals/genotype (histology; per animal (mean ±SEM): pS129 αS: TgA53T (0.180 ±0.073), TgA53T/mTau^-/-^ (0.172 ±0.034); Iba1: nTg (3.199 ±0.569), TgA53T (3.216 ±0.175), TgA53T/mTau^-/-^ (3.134 ±0.384); GFAP: nTg (4.053 ±0.307), TgA53T (4.028 ±0.380), TgA53T/mTau^-/-^ (3.579 ±0.202). Abbreviations: days post inoculation, dpi. Scale bars = 100 μm; error bars represent mean ± SEM


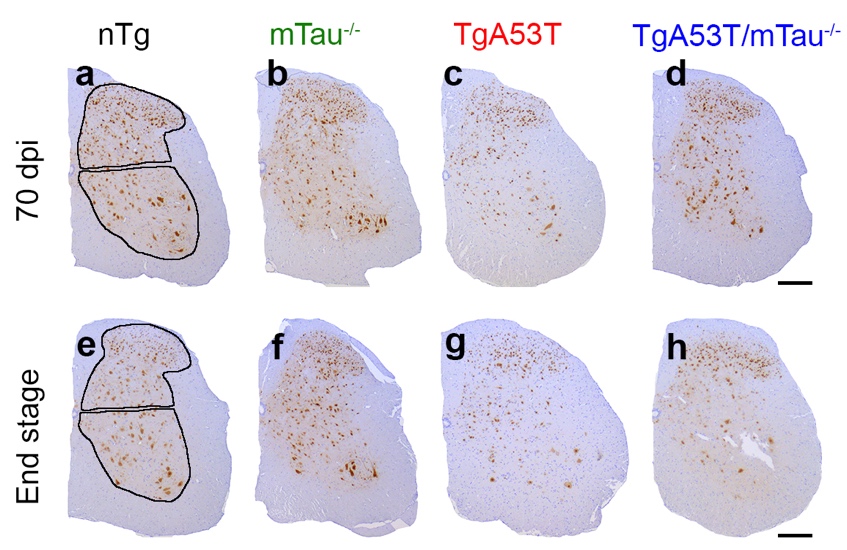


**Figure S11.** Representative low magnification images of 70 dpi and end stage spinal cord NeuN histology (see analysis in Fig. 6). Representative hemi-spinal cord images of 70 dpi (**a-d**), and end stage (**e-h**). The dorsal and ventral horn is outlined in nTg sections representing the respective regions of quantification. Scale bars = 250 μm


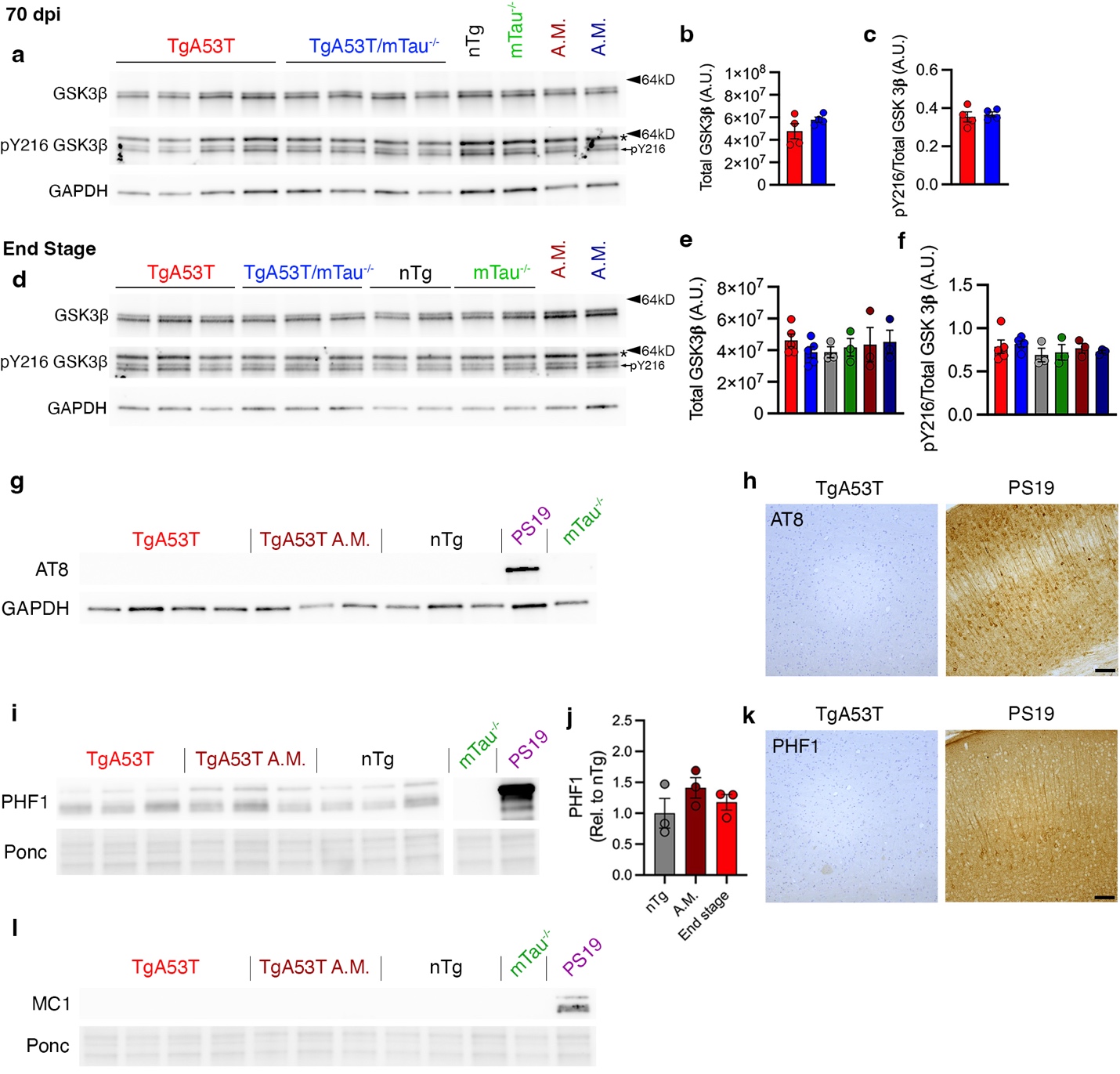
**Figure S12.** Analysis of GSK3β expression in 70 dpi (**a-c**) and end stage (**d-f**) spinal cords. **a-f** Expression of total GSK3β and pY216 GSK3β (active form) were analyzed by immunoblot analysis. The results show that neither total GSK3β or pY216 GSK3β are increased in the spinal cord of TgA53T mice and the levels were unaffected by tau expression. Evaluation of AT8 (**g; h** TgA53T – brainstem, PS19 - cortex), PHF1 (**i, j; k** TgA53T – brainstem, PS19 - cortex), and MC1 (**l**), show no pathological phosphorylated tau in spinal cord lysates of end stage TgA53T mice, as well as age-matched TgA53T compared to nTg, with 13-month-old transgenic P301S human tau (PS19) positive control, and mTau^-/-^ negative control mice. Pathological tau was also not observed after histological analysis of TgA53T end stage brainstem, compared to PS19 positive control. * - denotes GSK3α subunit band. All quantified bands were normalized to the respective GAPDH band. N = 3-5 animals/genotype. Abbreviations: Age-matched (non-injected), A.M.; arbitrary units, A.U. Error bars represent mean ± SEM. Scale bars = 100 μm.


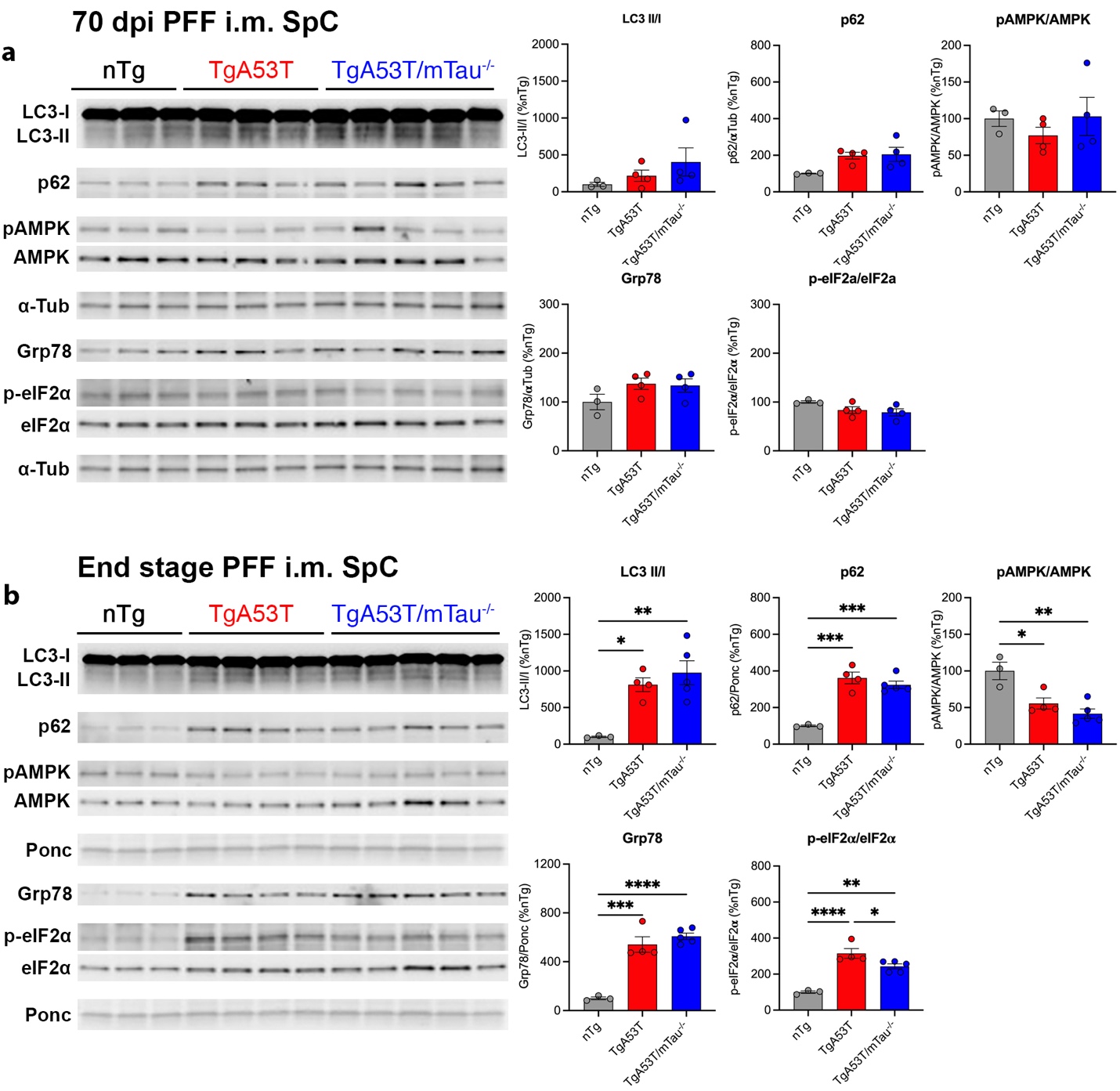
**Figure S13.** Endoplasmic reticulum stress and autophagy pathway protein clearance pathways deficits in TgA53T mice are not affected by tau expression. Immunoblot and quantitative analysis of endoplasmic reticulum stress and autophagy pathway proteins are unchanged in TgA53T mice at 70 dpi compared to nTg controls (**a**) but show dysfunction at end stage (**b**) in TgA53T spinal cords (SpC), which is independent of tau expression. One-way ANOVA with Tukey’s posthoc analysis (**b**): LC3 II/I: F_(2,9)_=10.55, p = 0.0044; p62: F_(2,9)_=29.85, p = 0.0001; pAMPK/AMPK: F_(2,9)_=12.41, p=0.0026; Grp78: F_(2,9)_=37.56, p<0.0001; p-eIF2α/eIF2α: F(2,9)=28.45, p=0.0001. p-eIF2α/eIF2α was significantly reduced in end stage TgA53T mice lacking tau (p = 0.0458). All quantified bands were normalized to the respective ponceau S total protein or α-tubulin bands. N = 3-5 animals/genotype. **p*<0.05, ***p*<0.01, ***p<0.001, ****p<0.0001; error bars represent mean ± SEM.


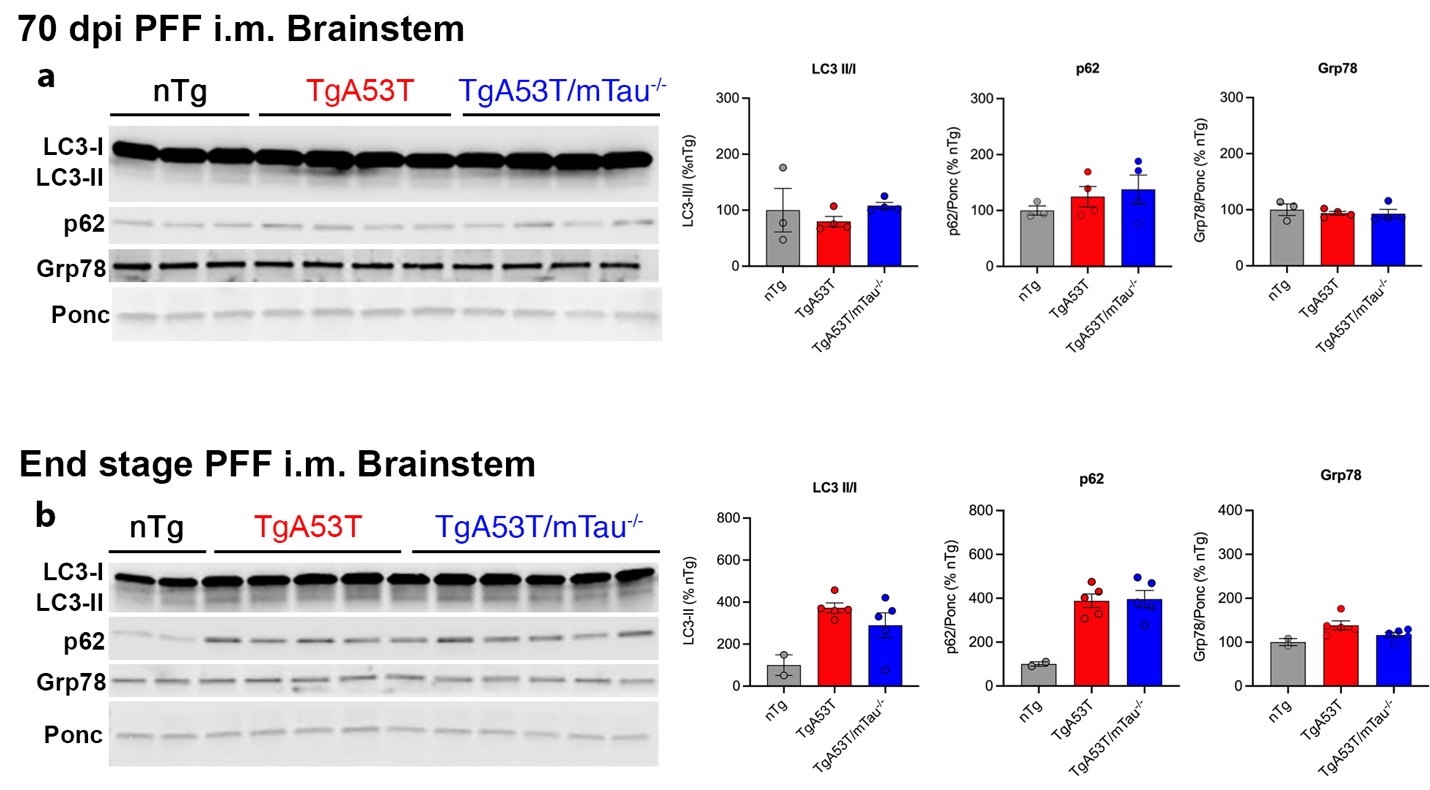


**Figure S14.** Endoplasmic reticulum stress and autophagy pathway protein clearance pathway analysis in 70 dpi and end stage brainstems. Immunoblot and quantitative analysis of endoplasmic reticulum stress (ERS) and autophagy pathway proteins are unchanged in brainstems of TgA53T mice compared to nTg controls at 70 dpi (**a**) and while autophagy related proteins are increased at end stage (**b**), ERS marker Grp78 is unchanged. All quantified bands were normalized to the respective ponceau S total protein. N = 2-5 animals/genotype. Error bars represent mean ± SEM

**
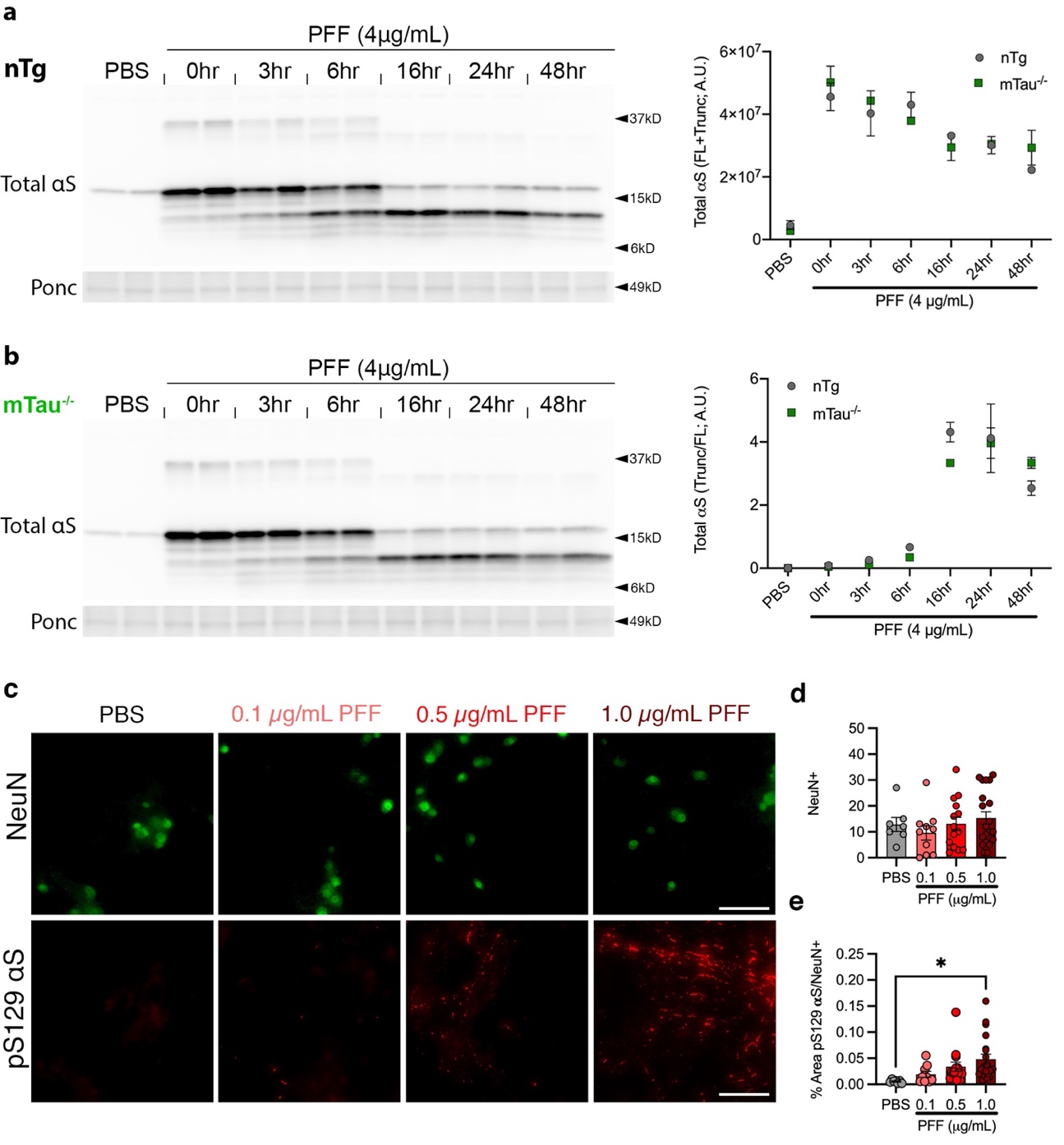
**

**Figure S15.** PFF uptake and processing in primary neurons is not affected by tau expression. **a, b** Primary hippocampal neurons were treated with αS PFF and internalized αS was analyzed via immunoblot analysis at various times. The results show that the initial acute uptake of αS PFF, as well as processing of full length αS to truncated form, is comparable in nTg and mTau^-/-^ neurons. Immunocytochemical analysis of NeuN+ neurons (**c** & **d**) and pS129 αS (**c** & **e**) following αS PFF treatment (0.1, 0.5, and 1.0 μg/mL) show lack of neuronal loss at 14 days post PFF treatment despite the presence of pS129 αS. All quantified bands were normalized to the respective ponceau S total protein. N = 2 genotype/timepoint (immunoblot), N = 7-20 randomly selected areas from 3 independent cultures. Abbreviations: phosphate buffered saline, PBS; full length, FL; truncated, trunc.; preformed fibril, PFF; arbitrary units, A.U.; Scale bars = 100 μm; error bars represent mean ± SEM


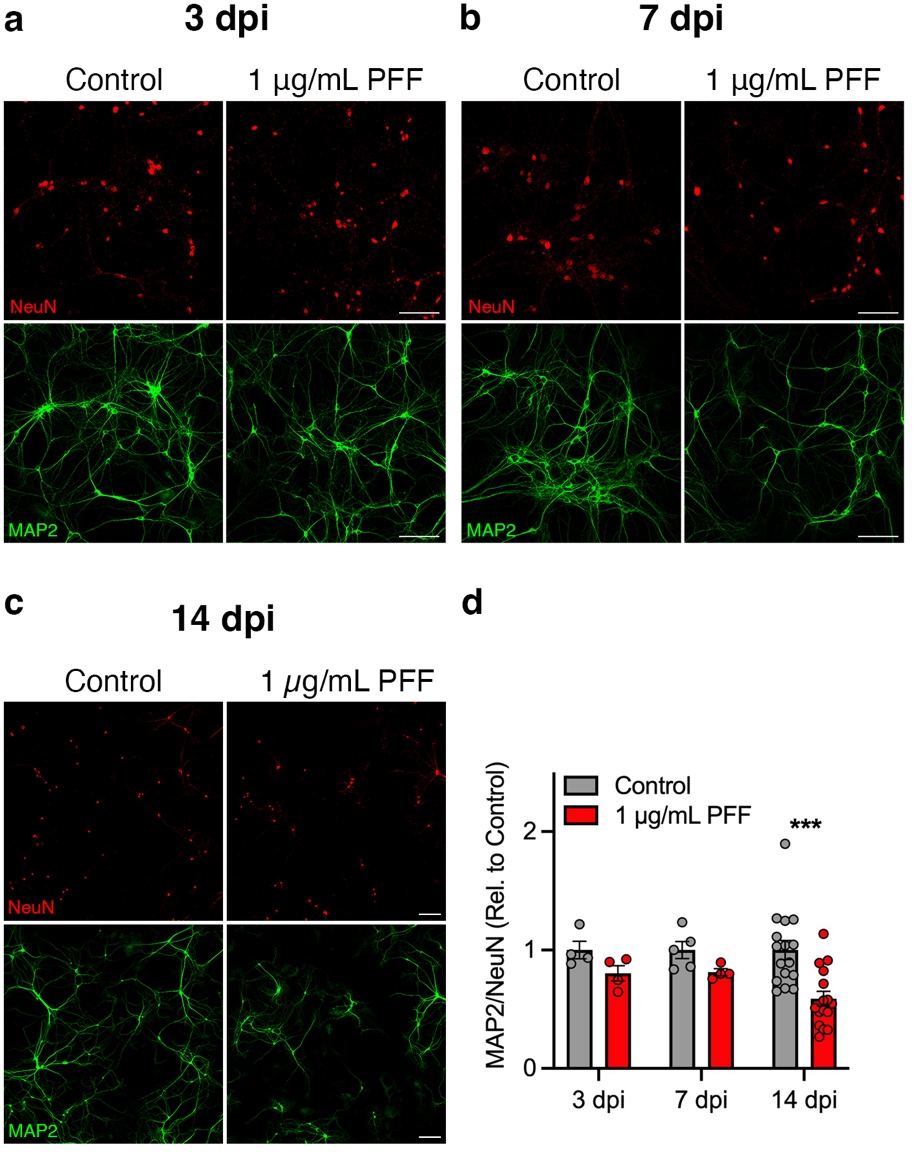


**Figure S16.** PFF leads to simplification of dendritic morphology after 14 dpi in nTg neurons *in vitro*. Primary hippocampal neurons were treated with either PBS, or 1 μg/mL of PFF and analyzed at 3 dpi (**a**), 7 dpi (**b**), or 14 dpi (**c**; data also represented in Fig. 8e & g). **d** PFF did not significantly affect neuronal morphology at 3 (p = 0.0937) and 7 dpi (p = 0.0669) but was significantly reduced at 14 dpi (p = 0.0003). N=4-16 randomly selected areas from 2-5 independent cultures. Abbreviations: days post inoculation, dpi; preformed fibril, PFF. Scale bars = 100 μm; error bars represent mean ± SEM
